# CRISPR–Cas9-enabled genetic disruptions for understanding ethanol and ethyl acetate biosynthesis in *Kluyveromyces marxianus*

**DOI:** 10.1186/s13068-017-0854-5

**Published:** 2017-06-24

**Authors:** Ann-Kathrin Löbs, Ronja Engel, Cory Schwartz, Andrew Flores, Ian Wheeldon

**Affiliations:** 1 Department of Chemical and Environmental Engineering, University of California, Riverside, 900 University Ave, Riverside, 92521 USA; 2 Mannheim University of Applied Sciences, Mannheim, Germany

**Keywords:** Acetate esters, Bioethanol, CRISPR–Cas9, Hemiacetal, Metabolic engineering, Thermotolerant yeast

## Abstract

**Background:**

The thermotolerant yeast *Kluyveromyces marxianus* shows promise as an industrial host for the biochemical production of fuels and chemicals. Wild-type strains are known to ferment high titers of ethanol and can effectively convert a wide range of C_5_, C_6_, and C_12_ sugars into the volatile short-chain ester ethyl acetate. Strain engineering, however, has been limited due to a lack of advanced genome-editing tools and an incomplete understanding of ester and ethanol biosynthesis.

**Results:**

Enabled by the design of hybrid RNA polymerase III promoters, this work adapts the CRISPR–Cas9 system from *Streptococcus pyogenes* for use in *K. marxianus*. The system was used to rapidly create functional disruptions to alcohol dehydrogenase (ADH) and alcohol-*O*-acetyltransferase (ATF) genes with putative function in ethyl acetate and ethanol biosynthesis. Screening of the *Km*ATF disrupted strain revealed that Atf activity contributes to ethyl acetate biosynthesis, but the knockout reduced ethyl acetate titers by only ~15%. Overexpression experiments revealed that *Km*Adh7 can catalyze the oxidation of hemiacetal to ethyl acetate. Finally, analysis of the *Km*ADH2 disrupted strain showed that the knockout almost completely eliminated ethanol production and resulted in the accumulation of acetaldehyde.

**Conclusions:**

Newly designed RNA polymerase III promoters for sgRNA expression in *K. marxianus* enable a CRISPR–Cas9 genome-editing system for the thermotolerant yeast. This system was used to disrupt genes involved in ethyl acetate biosynthesis, specifically *Km*ADH1–7 and *Km*ATF. *Km*Adh2 was found to be critical for aerobic and anaerobic ethanol production. Aerobically produced ethanol supplies the biosynthesis of ethyl acetate catalyzed by *Km*Atf. *Km*Adh7 was found to exhibit activity toward the oxidation of hemiacetal, a possible alternative route for the synthesis of ethyl acetate.

**Electronic supplementary material:**

The online version of this article (doi:10.1186/s13068-017-0854-5) contains supplementary material, which is available to authorized users.

## Background

The yeast *Kluyveromyces marxianus* is a potentially valuable host for industrial biochemical synthesis. Wild-type strains are known to have fast growth kinetics, thermotolerance to ~50 °C, the ability to metabolize a range of monomeric and dimeric C_5_ and C_6_ sugars, and strong fermentation pathways for ethanol production [1–3]. A high capacity for protein expression and secretion is also advantageous and has been exploited in bioprocesses for inulase and galactosidase production [4, 5]. Another industrially relevant phenotype is the native capacity of various strains to synthesize acetate esters. Ethyl acetate and other short-chain volatile esters are used as industrial solvents and as flavor and fragrance compounds; worldwide ethyl acetate demand is ~1.7 million tons per year [6, 7]. This demand is typically met by converting ethanol and acetate into ethyl acetate by Fisher esterification in reactive distillation processes [6, 8]. A recent bioprocessing pilot plant using *K. marxianus* produced ethyl acetate titers of 10.9 g L^−1^ from waste whey feeds with yields of 51.4%, thus providing a single-step alternative to the traditional process that relies on petroleum feedstocks (for acetate production) and multiple unit operations (ethanol fermentation, acetate production, and Fisher esterification) [8].

Metabolic synthesis of ethyl acetate and other short- and medium-chain esters can occur via a number of pathways [7]. In *Saccharomyces cerevisiae*, ester biosynthesis is primarily catalyzed by alcohol-*O*-acetyltransferase (Atf or AATase) activity that condenses acetyl-CoA with an alcohol to produce the corresponding ester [9, 10]. Double knockout of ATF1 and ATF2 in *S. cerevisiae* eliminates the synthesis of the medium-chain ester isoamyl acetate and reduces ethyl acetate production by 50% [11]. These enzymes localize to the endoplasmic reticulum and lipid droplets; this has been shown to be critical for high activity [12, 13]. Reverse esterase and alcohol dehydrogenase (Adh) activity may also contribute to acetate ester production. In *Candida utilis*, ethyl acetate is produced by Adh oxidation of a hemiacetal intermediate (the spontaneous product of ethanol and acetaldehyde) [14]. Adh1 from *S. cerevisiae* (*Sc*Adh1) and an Adh from *Neurospora crassa* are also known to exhibit hemiacetal oxidation activity [15, 16], a reaction that is thought to be involved in aldehyde detoxification under conditions of low NADH availability [7]. In *K. marxianus*, both Atf and reverse esterase activities have been identified, and Atf activity is thought to be primarily responsible for ethyl acetate biosynthesis [17, 18]. However, the metabolism of *K. marxianus* is less well understood than that of *S. cerevisiae*, and promiscuous Adh activity toward hemiacetal oxidation and the roles of different Adh enzymes in ester and alcohol metabolism are not yet completely understood.

Seven unique ADH genes have been previously identified in *K. marxianus*. *Km*ADH1, -2, -3, and -4 were identified as paralogs to *K. lactis* ADH genes, and recent transcriptional studies identified three additional ADH genes, *Km*ADH5, -6, and -7 [19–23]. Protein homology analysis revealed that *Km*Adh5 and -6 are similar to *Sc*Adh4 and -6, respectively [19, 23]. *Km*Adh7 has high protein homology to an Adh from the bacterium *Cupriavidus necator* [19]. Thus far, most studies have focused on *Km*ADH1–4, and the corresponding enzymes have been assigned to the group of zinc-dependent alcohol dehydrogenases that preferentially use NAD(H) over NADP(H) [21, 24]. The identified *K. marxianus* ADH genes along with *Km*ATF are summarized in Table 1.

**Table 1:** *Kluyveromyces marxianus* alcohol dehydrogenases (Adh) and alcohol-*O*-acetyltransferase (Atf)

Name	Alternative names	Putative function	Homology (identity, similarity)	Size	Reference	Gene product
*Km*Adh1	–	Ethanol production	*Kl*Adh1 (85.2, 92.0%) *Kl*Adh2 (86.5, 92.0%) *Sc*Adh2 (79.4, 88.0%) *Sc*Adh1 (76.3, 84.8%)	348aa	[19–24]	BAO40126.1
*Km*Adh2	–	Ethanol production	*Sc*Adh1 (86.0, 91.4%) *Kl*Adh2 (83.7, 91.1%) *Kl*Adh1 (86.0, 90.9%) *Sc*Adh2 (84.5, 90.3%)	348aa	[19–24]	BAO40244.1
*Km*Adh3	–	Use of nonfermentable carbon sources	*Kl*Adh3 (91.2, 94.4%) *Sc*Adh3 (77.5, 87.0%)	375aa	[19–24]	BAO42617.1
*Km*Adh4	ADH4b [19]	Ethanol detoxification	*Kl*Adh4 (90.8, 93.9%) *Sc*Adh3 (78.9, 87.6%)	379aa	[19–24]	BAO38616.1
*Km*Adh5	ADHb [20], ADH4a [19]	Unknown	*Sc*Adh3 (65.2, 78.3%)	418aa	[19, 20, 23]	BAO38463.1
*Km*Adh6		Unknown	*Sc*Adh6 (62.0, 76.4%) *Sc*Adh7 (58.8, 74.3%)	366aa	[19, 20, 23]	BAO42650.1
*Km*Adh7	Adha [20], adh [19]	Unknown	*Acinetobacter equi* adh (67.2, 81.4%) Snodgrassella alvi adh (67.4, 80.9%) *Cupriavidus necator* adh (30.8, 50.2%)	386aa	[19, 20]	BAO40648.1
*Km*Atf	Atf1 [19]	Unknown	*Kl*Atf (56.0, 75.8%) *Sc*Atf2 (31.7, 53.1%) *Sc*Atf1 (35.0, 52.5%)	515aa	[19, 20]	BAO39498.1

To enable gene disruption studies and identify the roles of *Km*ADH1–7 and *Km*ATF in ethyl acetate and ethanol biosynthesis, we adapted the type II CRISPR–Cas9 system from *S. pyogenes* for genome editing in *K. marxianus*. *K. marxianus* is known to have a high capacity for DNA repair by nonhomologous end joining (NHEJ), which can limit the rapid generation of targeted knockout libraries by traditional genome manipulation techniques [2, 25]. In this work, we used a hybrid promoter strategy to express single-guide RNAs (sgRNAs) that target Cas9 endonuclease to *Km*ADH1–7 and *Km*ATF for functional disruption in the CBS 6556 strain of *K. marxianus* [26, 27]. Transcriptional and functional analysis of the disruption library revealed the critical role of *Km*ADH2 in ethanol production, and that disruption results in acetaldehyde accumulation. In addition, analysis of the knockout strains coupled with overexpression studies revealed novel hemiacetal activity toward ethyl acetate synthesis from *Km*Adh7 and showed that *Km*Atf has activity toward the condensation of acetyl-CoA and ethanol.

## Results

### Thermotolerance and ethyl acetate biosynthesis in *K. marxianus* CBS 6556

**Fig. 1:**
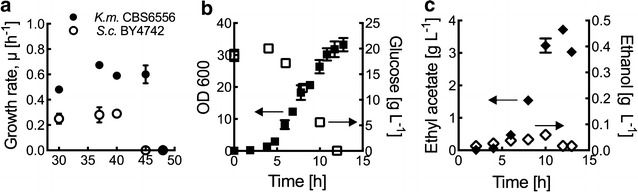
Ethyl acetate and ethanol production in thermotolerant *K. marxianus* CBS 6556. **a** Specific growth rates of *K. marxianus* CBS 6556 and *S. cerevisiae* BY4742 were determined by shake flask culturing at temperatures of 30, 37, 40, 45, and 48 °C. **b** Aerated bioreactor growth and glucose consumption of *K. marxianus* CBS 6556 at 60% dissolved oxygen and 37 °C. **c** *K. marxianus* CBS 6556 production of ethyl acetate and ethanol during the bioreactor experiments shown in **b**. Data points represent the arithmetic mean of three biological replicates, and the *error bars* represent the standard deviation

Various strains of *K. marxianus* have been shown to have fast growth kinetics at temperatures above 40 °C [2, 28]. For example, the strain CBS 6556 exhibits high growth rates at 45 °C (0.60 ± 0.07 h^−1^; Fig. 1a). High growth rates were also observed at lower temperatures and were nearly twice that of the model yeast *S. cerevisiae* BY4742. We have previously identified CBS 6556 as a high producer of ethyl acetate and here demonstrate the growth-associated production of 3.72 ± 0.06 g L^−1^ from the wild type in a controlled aerated bioreactor (Fig. 1b, c). Selectivity toward ethyl acetate was high, as only a limited amount of ethanol (60 ± 11 mg L^−1^) was coproduced. Given the fast growth kinetics, thermotolerance to 45 °C, and the high capacity to synthesize ethyl acetate, we selected CBS 6556 as a model *K. marxianus* strain to further understand the roles of *Km*ADHs and *Km*ATF in ethyl acetate and ethanol biosynthesis.

### Ethyl acetate synthesis activity in *K. marxianus*

**Fig. 2:**
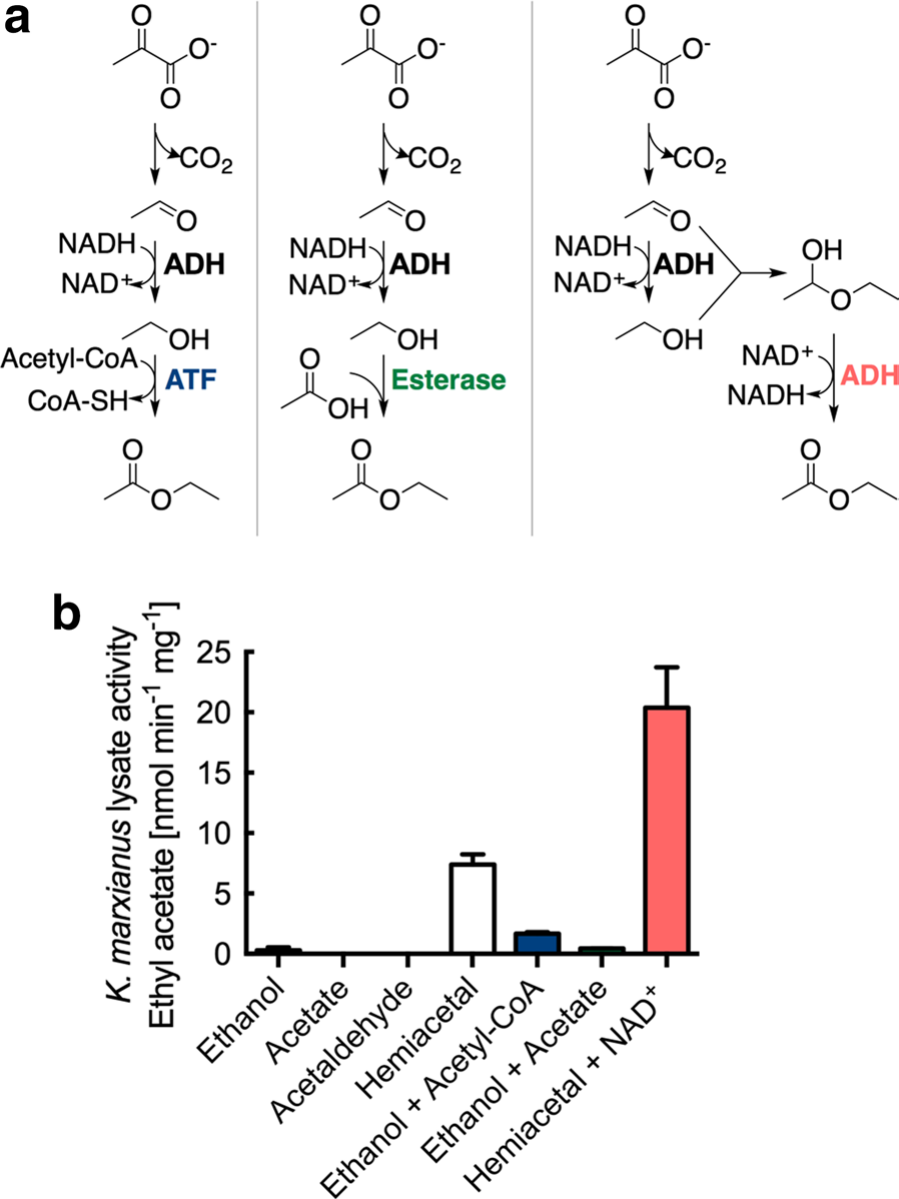
Ethyl acetate biosynthetic pathways and synthesis activities in *K. marxianus*. **a** Ethyl acetate biosynthesis via alcohol-*O*-acetyltransferase (Atf; *left*), reverse esterase activity (*middle*), and by alcohol dehydrogenase (Adh) oxidation of hemiacetal (*right*). **b** *K. marxianus* CBS 6556 lysate assays for ethyl acetate production. Reactions were accomplished with 100 µg of lysate protein buffered to pH 7.2 with 100 mM potassium phosphate and 30 °C. Data points represent the arithmetic mean of three biological replicates, and the *error bars* represent the standard deviation

Three metabolic pathways are known to produce ethyl acetate: (1) the condensation of ethanol and acetyl-CoA; (2) the reverse esterase synthesis of ethyl acetate from ethanol and acetate; and (3) the oxidation of hemiacetal (Fig. 2a) [7]. Similar to other yeasts, ethanol is synthesized by the decarboxylation of pyruvate and the Adh-mediated reduction of acetaldehyde to ethanol. To identify activities toward ethyl acetate that are present in *K. marxianus*, we conducted a series of cell lysate assays supplemented with appropriate enzyme substrates (Fig. 2b). The addition of hemiacetal and NAD^+^ cofactor to cell lysates synthesized 20.4 ± 3.3 nmol min^−1^ mg^−1^ of ethyl acetate, while the addition of ethanol and acetyl-CoA produced 1.7 ± 0.1 nmol min^−1^ mg^−1^ (rates reported as per mg of lysate protein). A control sample of hemiacetal without NAD^+^ produced 7.4 ± 0.8 nmol min^−1^ mg^−1^, likely due to cofactor present in the cell lysate. Reverse esterase activity was significantly limited (0.44 ± 0.02 nmol min^−1^ mg^−1^), and control assays with only ethanol, acetate, and acetaldehyde did not produce measurable amounts of ethyl acetate. Combined together, the assays suggest that ethyl acetate may be produced from one or both of the Atf- and Adh-dependent pathways.

### CRISPR–Cas9-mediated gene disruptions in *K. marxianus*

**Fig. 3:**
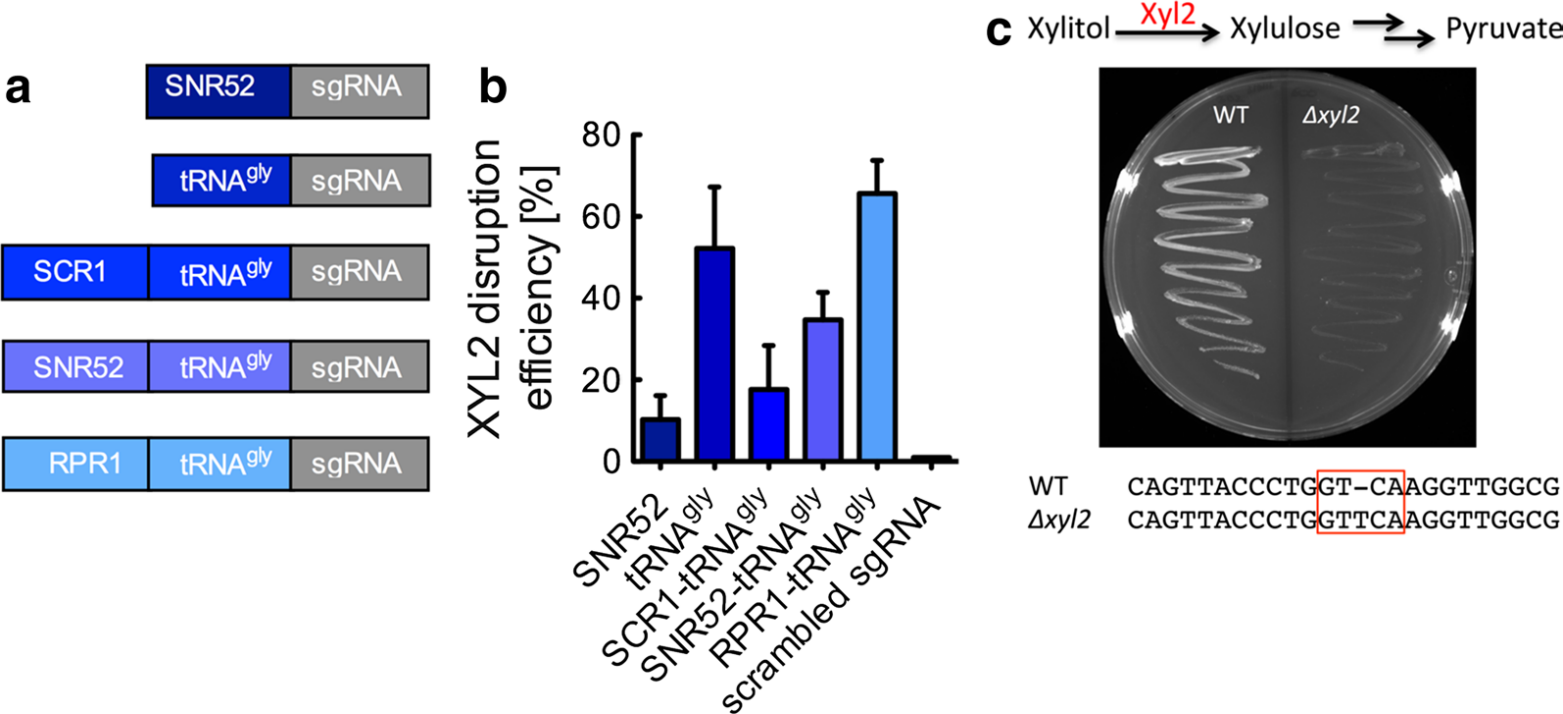
CRISPR–Cas9 genome editing in *K. marxianus*. **a** The design of native and hybrid RNA polymerase III promoters for sgRNA expression. The 20 bp sgRNA targeting sequence targets xylitol dehydrogenase (XYL2). **b** CRISPR–Cas9-induced disruption efficiencies of the XYL2 gene. Cultures were transformed with a single vector expressing codon-optimized Cas9 and the sgRNA. Cultures were grown on selective media for 2 days prior to screening on xylitol supplemented agar media. Data points represent the arithmetic mean of 30 colonies randomly selected from three different transformations. *Error bars* represent the standard deviation of the samples. **c** XYL2-disrupted strains were restreaked on xylitol-containing agar plates and screened for loss of growth. Gene disruption was confirmed by sequencing

CRISPR–Cas9 genome-editing systems function by targeting the Cas9 endonuclease to specific loci in the genome through complexation with guide RNAs (sgRNAs). sgRNA expression is often limiting to Cas9 function [27, 29]. To address this potential problem, we designed a series of native and hybrid RNA polymerase III promoters for sgRNA expression, including SNR52, tRNA^Gly^, and fusions of SNR52-tRNA^Gly^, SCR1-tRNA^Gly^, and RPR1-tRNA^Gly^ (Fig. 3a). We targeted xylitol dehydrogenase (XYL2) for promoter testing because successful disruption can be coupled to a phenotype that is easily screened, i.e., the loss of growth on xylitol supplemented agar media plates (Fig. 3c). XYL2 disruption efficiencies were determined by restreaking a minimum of 30 colonies that were subjected to mutation by CRISPR–Cas9 onto solid media plates with xylitol as the sole carbon source. Disruption efficiency was found to be promoter-dependent (Fig. 3b). The highest efficiency, 66 ± 8%, was achieved with the RPR1-tRNA^Gly^ promoter, while tRNA^Gly^ achieved 52 ± 15% disruption efficiency. The SNR52, SNR52-tRNA^Gly^, and SCR1-tRNA^Gly^ promoters were less successful, resulting in disruption efficiencies of 10 ± 6, 35 ± 7, and 18 ± 11%, respectively. Gene disruption was also found to be sgRNA sequence-dependent (Additional file 1: Table S1), and a scrambled sgRNA did not produce a loss of xylitol dehydrogenase function (Fig. 3b).

With a functional CRISPR–Cas9 system in hand, we created a library of mutant CBS 6556 strains with functional disruptions to *Km*ADH1–7 and *Km*ATF. In each case, sgRNA design was accomplished using a previously published sgRNA scoring algorithm to identify high-scoring sgRNAs upstream of each enzyme’s putative active site [30]. The protocol for creating and screening genetic disruptions by CRISPR–Cas9 is schematically described in Additional file 1: Figure S1. The final steps of the protocol include quantifying the rate of gene disruption, which was determined by amplifying the gene of interest from the genome, and sequencing the PCR product to identify indels. The indel success rates, which ranged from 10 to 67%, are presented in Table 2. Importantly, the mutant strains used in subsequent experiments were cured of the CRISPR–Cas9 plasmid, and the gene of interest was sequenced to identify the specific mutations in each gene (Table 2). In each case, the indel created a genetic frameshift mutation that results a premature stop codon, thus functionally disrupting the gene.

**Table 2:** *Km*ADH and *Km*ATF CRISPR–Cas9 target sequences and knockout efficiencies

Target	SgRNA	Gene disruption	Indel success
*Km*ADH1	AGACTTCAAAGCCTTGTACA	WT CCGTGTACAAGGCTTTGAAG KO CCG--------GCTTTGAAG	2/10
*Km*ADH2	GGTACCAGCTGGGATGTGAG	WT AAGCCGCTCACATCCCAGCT KO AAGCCGCT-----CCCAGCT	4/30
*Km*ADH3	GCTATTCCAGAAAAGCAAAA	WT CAGAAAAGCAAAAGGGTGTT KO CAGAAAA-----AGGGTGTT	2/10
*Km*ADH4	GCCATCCCAGAATCCCAAAA	WT TCCCAGAATCCC-AAAAGGG KO TCCCAGAATCCCAAAAAGGG	4/10
*Km*ADH5	ATGGTCTTGAAAGAACACAA	WT GGTCTTGAAAGAA-CACAAG KO GGTCTTGAAAGAACCACAAG	1/10
*Km*ADH6	GTACCACCACCGCAAAGTAG	WT CTACTTTGCGGTGGTGGTA KO CTACTT-GCGGTGGTGGTAC	2/10
*Km*ADH7	GCTTGAGCTGAGAGATTGAT	WT AAGTCCCATCAA-TCTCTCA KO AAGTCCCATCAAATCTCTCA	3/5
*Km*ATF	ATATAGTCTTCGGCAACACC	WT CGGTGTTGCCGAAGACTAT KO CGGTG--GCCGAAGACTATA	4/10

### The effect of *Km*ATF disruption on ethyl acetate biosynthesis

**Fig. 4:**
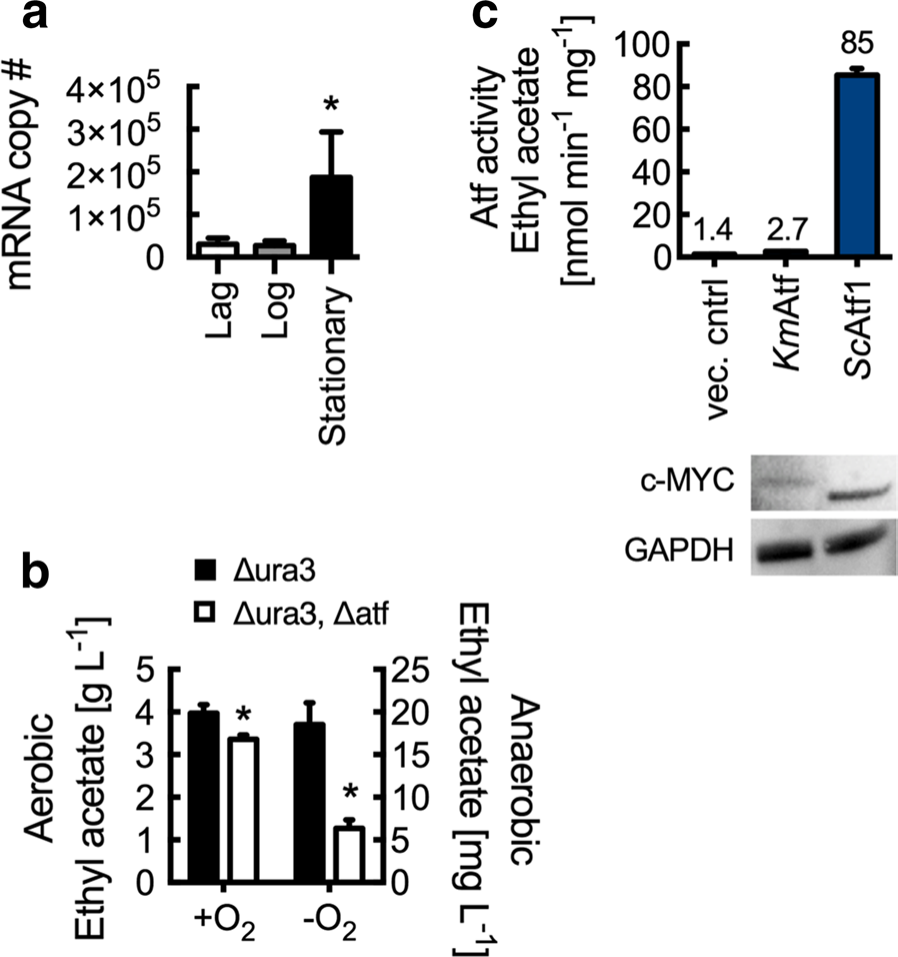
Ethyl acetate biosynthesis in *K. marxianus* by alcohol-*O*-acetyltransferase. **a** RT-qPCR analysis of wild-type *K. marxianus* ATF transcript levels at lag (5 h), log (10 h), and stationary (18 h) phases. mRNA copy number represents total number of transcripts for 5 ng of isolated RNA. **b** *Km*ATF disruption reduces ethyl acetate production in both aerobic and anaerobic conditions. Cultures were grown at 37 °C for 10 h (aerobic) and 14 h (anaerobic). **c** Lysate Atf activity from *S. cerevisiae* with overexpressed *Km*ATF and *Sc*ATF1. Rates are reported per mg of lysate protein. Enzyme expression was confirmed by Western blot analysis using anti-c-MYC and anti-GAPDH antibodies. *Bars* and *error bars* represent the arithmetic mean and standard deviation of triplicate biological samples. Statistical significance (*P* < 0.05) is indicated by *asterisk* and was determined using a *T* test

Figure 1b, c as well as previous *K. marxianus* studies show that ethyl acetate production is growth-associated, as such *Km*ATF transcript levels were examined at lag, log, and stationary phase [1, 31]. Reverse transcription quantitative PCR (RT-qPCR) analysis showed that under aerobic conditions (the conditions required for high ethyl acetate production) *Km*ATF is upregulated during stationary phase (Fig. 4a). Anaerobically, *Km*ATF followed a growth-associated expression pattern (Additional file 1: Figure S2). CRISPR–Cas9 disruption of the gene produced a 15% reduction in ethyl acetate during aerobic growth and a 66% reduction under anaerobic conditions, suggesting that *Km*Atf has activity toward ethyl acetate biosynthesis (Fig. 4b). The effect of the gene disruption when cultured aerobically on synthetic minimal media was not statistically significant (Additional file 1: Figure S3). The alcohol-*O*-acetyltransferase activity of *Km*Atf was confirmed in lysate experiments with overexpression in *S. cerevisiae* (Fig. 4c); however, *Km*Atf (2.7 ± 0.1 nmol min^−1^ mg^−1^) was found to be less active than *Sc*Atf1 (85.4 ± 3.1 nmol min^−1^ mg^−1^). Western blot analysis of the *S. cerevisiae* lysates used in the assay confirmed protein expression and suggested that some of the difference in activity was due to reduced enzyme expression (Fig. 4c).

### The effect of *Km*ADH1–7 disruptions on ethyl acetate and ethanol biosynthesis

**Fig. 5:**
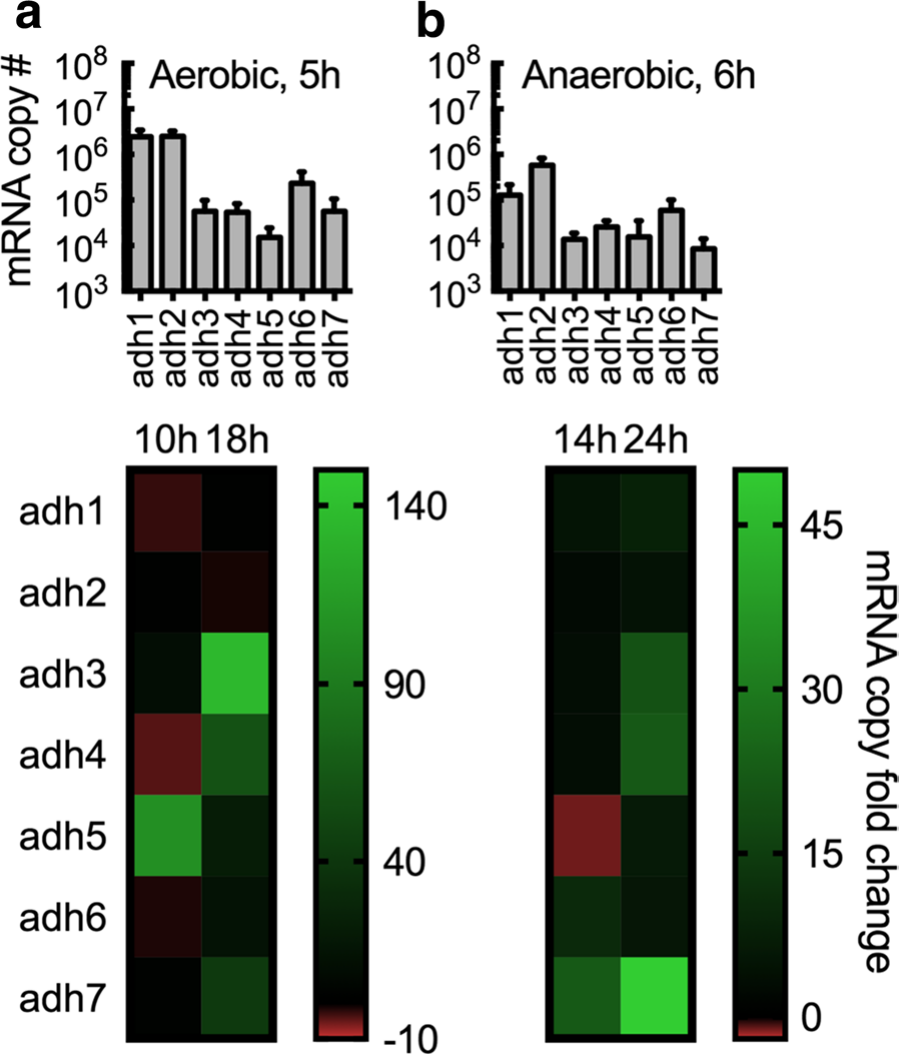
Transcriptional analysis of *Km*ADH expression in wild-type *K. marxianus* CBS 6556. mRNA copy numbers at lag, log, and stationary phases of aerobic (**a**) and anaerobic (**b**) growth were determined by RT-qPCR. The *top panels* of **a** and **b** show the total mRNA copy numbers of *Km*ADH1–7 expressed in *K. marxianus* strain CBS 6556 at 5 h for aerobic cultures and 6 h for anaerobic cultures. mRNA copy number represents total number of transcripts for 5 ng of isolated RNA. The heat maps below show the mRNA copy fold change of the individual genes at log (10 and 14 h) and stationary phases (18 and 24 h) compared with the expression at lag phase (5 and 6 h). *Bars* and *error bars* represent the arithmetic mean and standard deviation of triplicate biological samples

To determine the effects of *Km*ADH1–7 on ethanol and ethyl acetate biosynthesis, wild-type CBS 6556 and mutant strains were cultured, monitoring growth, gene expression, ethanol, ethyl acetate, and acetaldehyde production. The transcriptional patterns of the *Km*ADHs in wild-type CBS 6556 are shown in Fig. 5. Transcriptional analysis was conducted aerobically at 5, 10, and 18 h, corresponding to lag, log, and stationary phases, respectively (Fig. 5a). *Km*ADH1 and -2 were highly expressed after 5 h of aerobic growth and transcript levels remain consistent after 10 and 18 h. Transcript levels of *Km*ADH3 and -4 at 5 h were significantly lower in comparison with *Km*ADH1 and -2, but were upregulated during stationary phase. Analysis of *Km*ADH5 revealed the lowest initial expression level, with increased expression after 10 h of aerobic culture. *Km*ADH6 expression remained consistent through each growth phase, and *Km*ADH7 increased expression as cells entered stationary phase, both of which maintained initial levels lower than the highly expressed *Km*ADH1 and -2. For the analysis of anaerobic expression, cultures were grown for 6, 14, and 24 h to lag, log, and stationary phases, respectively (Fig. 5b). *Km*ADH2 expression was the highest during all of the growth stages, indicating its importance in *K. marxianus* metabolism. Similar to *Km*ADH2, *Km*ADH1 was highly expressed throughout culture growth. *Km*ADH3 and -4 showed increased expressions at stationary phase, *Km*ADH6 and *Km*ADH7 expressions increased upon reaching stationary phase, and *Km*ADH5 expression was insensitive to growth phase.

With respect to cell growth, *Km*ADH1–7 and *Km*ATF disruptions had no effect on cell growth under aerobic conditions (Additional file 1: Figure S4). Under anaerobic conditions, ethanol fermentation pathways are significantly upregulated and are necessary for cell redox balance. As expected, *Km*ADH1–7 and *Km*ATF disruptions affected growth (Additional file 1: Figure S5). More specifically, disruptions of *Km*ADH2 and -4 resulted in growth rates of 0.25 ± 0.05 and 0.23 ± 0.08 h^−1^ compared with 0.36 ± 0.10 h^−1^ of CBS 6556 at 37 °C.

**Fig. 6:**
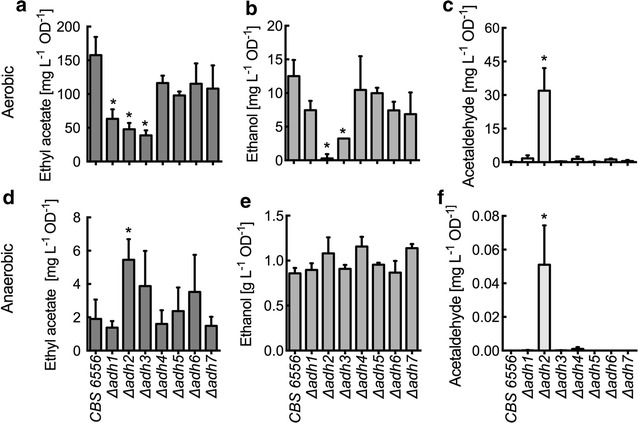
Ethyl acetate, ethanol, and acetaldehyde production in *K. marxianus* CBS 6556 and ADH disruption strains. **a**–**c** Aerobic production of ethyl acetate, ethanol, and acetaldehyde production per cell density after 10 h of growth in YM media at 37 °C. **d**–**f** Anaerobic production of ethyl acetate, ethanol, and acetaldehyde production per cell density after 14 h of growth. *Bars* and *error bars* represent the arithmetic mean and standard deviation of triplicate biological samples

Metabolite analysis revealed that *Km*ADH1, -2, and -3 mutants exhibited reduced ethyl acetate production compared with the wild-type CBS 6556 strain for aerobic cultures (Fig. 6a). Mutations to *Km*ADH1–3 resulted in specific ethyl acetate titers of 63.3 ± 14.1 mg L^−1^ OD^−1^, 47.9 ± 9.4 mg L^−1^ OD^−1^ and 38.8 ± 7.4 mg L^−1^ OD^−1^, compared with wild-type CBS 6556, which produced 157.8 ± 26.9 mg L^−1^ OD^−1^. Reduced ethanol production accompanied the loss of ethyl acetate biosynthesis (Fig. 6b). Disruption of *Km*ADH1 produced 7.5 ± 1.4 mg L^−1^ OD^−1^ of ethanol, while functional disruption to *Km*ADH2 and -3 produced only 0.28 ± 0.63 mg L^−1^ OD^−1^ and 3.2 ± 0.01 mg L^−1^ OD^−1^, respectively. In the case of *Δadh2*, an increase in acetaldehyde accumulation was also observed (31.9 ± 10.1 mg L^−1^ OD^−1^; Fig. 6c). These results suggest that *Km*ADH2 is critical to the supply of ethanol for aerobic ethyl acetate biosynthesis. Less significant changes in ethyl acetate, ethanol, and acetaldehyde were observed with disruptions to *Km*ADH4, -5, -6, and -7. It should be noted that the URA3-disrupted strain showed decreased ethyl acetate production of 67.9 ± 38.4 mg L^−1^ OD^−1^ compared with the wild type (Additional file 1: Figure S6). Figure 6d–f presents the volatile metabolite analysis for anaerobically grown cultures. While *Km*ADH1–7 disruption did not reduce overall ethanol production, acetaldehyde accumulation (0.05 ± 0.02 mg L^−1^ OD^−1^) was again observed in the *Δadh2* strain (Fig. 6e, f). Furthermore, *Km*ADH2 disruption led to a significant increase of anaerobic ethyl acetate production from 1.91 ± 1.16 mg L^−1^ OD^−1^ in the wild type to 5.46 ± 1.23 mg L^−1^ OD^−1^ in the disrupted strain (Fig. 6d). Note that (1) anaerobic ethanol production was ~50-folds greater than that was observed in aerobic cultures, and (2) aerobic ethyl acetate production was ~25-folds greater than that was observed anaerobic cultures.

### *Km*Adh activity toward ethyl acetate formation from hemiacetal

**Fig. 7:**
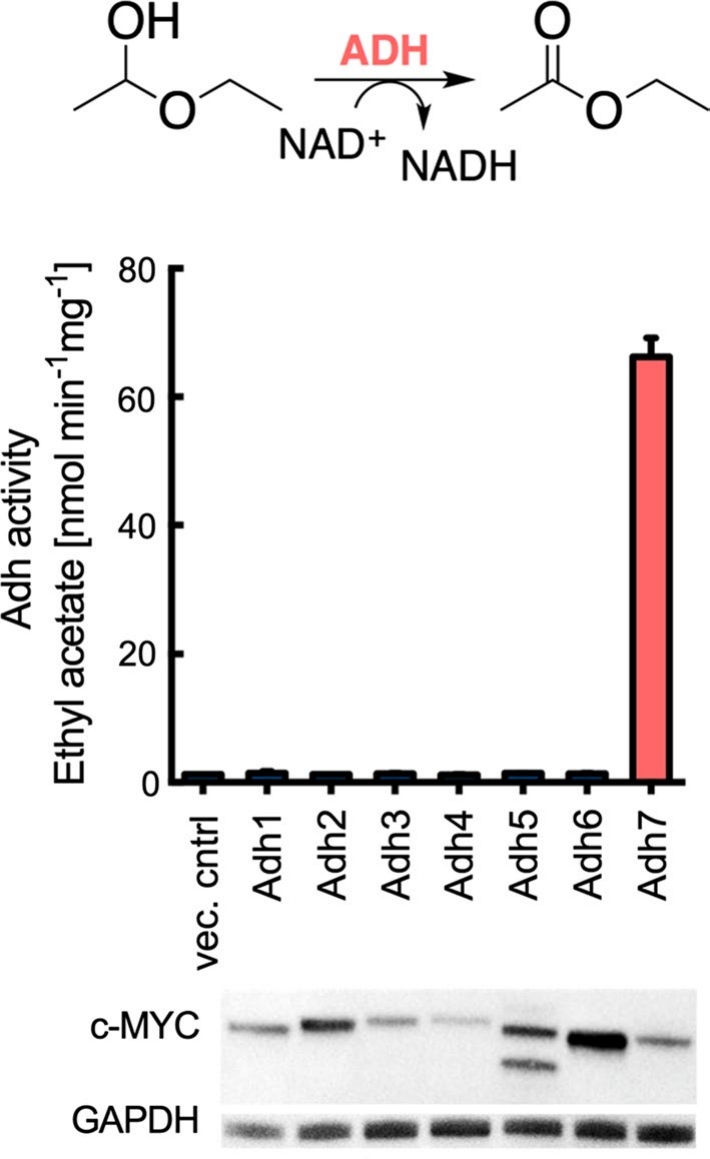
Hemiacetal oxidation activity of *K. marxianus* alcohol dehydrogenases. *Km*ADH1–7 were heterologously overexpressed in *S. cerevisiae* BY 4742. Cell lysate assays showed that *Km*Adh7 exhibits activity toward synthesis of ethyl acetate from hemiacetal. Enzyme expression was confirmed by Western blot using anti-c-MYC and anti-GAPDH antibodies. *Bars* and *error bars* represent the arithmetic mean and standard deviation of triplicate samples

Because the *Km*ADH1–7 disruption library did not reveal clear gene candidates for ethyl acetate production, each ADH gene was separately overexpressed in *S. cerevisiae* to facilitate lysate enzyme assays. *Sc*Atf1 and -2 are known to be suppressed by oxygen; therefore, the *S. cerevisiae* lysates provided a cell lysate background that lacks the capacity to produce ethyl acetate [32]. Western blot analysis of C-terminal MYC-tag modified enzymes confirmed the overexpression of *Km*Adh1, -2, -5, -6 and -7 (Fig. 7). *Km*Adh3 and -4 were also expressed but at reduced levels. *S. cerevisiae* lysates overexpressing each to the enzymes were incubated with hemiacetal and NAD^+^ cofactor, and the reactions were allowed to continue for 30 min. Notably, *Km*Adh7 produced ethyl acetate at a rate of 66.3 ± 2.9 nmol min^−1^ mg^−1^ of lysate protein. No other overexpressed enzymes produced measurable amounts of ethyl acetate. Thus far, the function of *Km*Adh7 has not been described, and the result presented here suggests hemiacetal activity toward the biosynthesis of ethyl acetate.

## Discussion

The CBS 6556 strain of *K. marxianus* is thermotolerant and exhibits fast growth kinetics at 45 °C (Fig. 1a). It ferments high titers of ethanol under anaerobic conditions, [2] and aerobically produces significant amounts of the short-chain volatile ester ethyl acetate (Fig. 1c). CBS 6556 and other strains of *K. marxianus* also have the ability to metabolize various C_5_, C_6_, and C_12_ carbon sources [1]. Collectively, these characteristics are useful for industrial-scale bioprocessing: high-temperature bioreactors can minimize the need for aseptic conditions, diverse carbon sources allow for the use of the lowest-cost sugars, and the high titer production of volatile compounds can facilitate low-cost product separation through distillation and stripping [33].

Despite these many advantages, *K. marxianus* strain development has been limited in comparison with the model yeast *S. cerevisiae* because genome-editing tools and stable heterologous expression systems developed for *K. marxianus* are limited [25, 34–36]. Enabled by hybrid RNA polymerase III promoters for sgRNA expression, we adopted the CRISPR–Cas9 system from *S. pyogenes* for *K. marxianus* genome editing (Fig. 3). This system was necessary to create a library of strains with disruptions to genes with suspected function in ethyl acetate and ethanol metabolism including *Km*ADH1–7 and *Km*ATF (Table 1). The main observations stemming from our CRISPR–Cas9 experiments are that (1) the RPR1-tRNA^Gly^ hybrid promoter achieved the highest knockout efficiencies (Fig. 3b), and (2) that gene disruption efficiency was found to be highly dependent on gene target and sgRNA sequence (Additional file 1: Table S2). We have previously observed a similar result for hybrid sgRNA promoter design in the yeast *Yarrowia lipolytica*, and the sequence dependency of sgRNAs has been described in both human and mouse systems [27, 30].

The widespread adoption of type II CRISPR–Cas9 technologies for genome editing has made less genetically tractable organisms more accessible [26, 27]. These tools are particularly useful in organisms where NHEJ prevails over DNA repair by homologous recombination and when genomes are diploid [37, 38]. For example, CRISPR–Cas9 genome editing has recently been demonstrated in the yeasts *Y. lipolytica*, *S. pompe*, *P. pastoris*, *K. lactis*, *C. albicans*, and *S. cerevisiae* [26, 27, 37–42]. Many of these examples focus on tool development for genome and metabolic engineering, including standardized and multiplexed methods for heterologous gene integration [26, 38, 41, 42]. The other examples use the genome-editing system, as we have done here, to study the effects of genetic disruptions on cell phenotypes and metabolism [37, 39, 40]. To our knowledge, these types of studies have not yet been accomplished in *K. marxianus*.

The *∆adh1*–*7* and *∆atf* strains of *K. marxianus* CBS 6556 were created to investigate the effects of these genes on ethyl acetate and ethanol biosynthesis. Our first experiments toward this goal helped identify the targeted knockouts. Specifically, *K. marxianus* lysate assays showed ethyl acetate biosynthesis from both Atf- and Adh-catalyzed reactions (Fig. 2). Atf activity was observed when lysates were supplemented with ethanol and acetyl-CoA, while Adh activity was observed when supplementing with hemiacetal and NAD^+^ cofactor. These results are in contrast to prior reports that describe Atf and reverse esterase activities as the only reactions responsible for ethyl acetate biosynthesis in *K. marxianus* DSM 5422 and other strains [17, 18]. In the CBS 6556 strain, we found no reverse esterase activity, but did identify Atf and Adh activities as possible routes for ethyl acetate production. On the basis of these results, *∆adh1*–*7* and *∆atf* strains of *K. marxianus* CBS 6556 were created to investigate the effects of these genes on ethyl acetate and ethanol biosynthesis.

Functional disruption of *Km*ATF resulted in the statistically significant reduction of ethyl acetate biosynthesis under both aerobic and anaerobic conditions (Fig. 4b). It is important to note here that ethyl acetate production was ~100-fold higher when cells were grown aerobically. Ethyl acetate synthesis through *Km*Atf activity was further confirmed in *S. cerevisiae* lysate assays containing heterologously overexpressed enzyme (Fig. 4c). Previous studies on *K. lactis* Atf (*Kl*Atf), which is most closely related to *Km*Atf, revealed limited activity toward ethyl acetate in comparison with *Sc*Atf1, a result that was also observed in our studies of *Km*Atf (Fig. 4c) [13, 43, 44]. Taken together, these results suggest that *Km*Atf activity contributes in part to ethyl acetate biosynthesis in *K. marxianus*, but that other alcohol-*O*-acetyltransferases and/or other metabolic pathways are also responsible.

It is well understood that ethanol production in yeast is accomplished by the Adh-catalyzed reduction of acetaldehyde. Various Adh enzymes have also been shown to catalyze ethyl acetate synthesis through the oxidation of hemiacetal (Fig. 2a) [7]. RT-qPCR analysis of the genes found to be most relevant to ethyl acetate production showed that *Km*ADH2 was constitutively expressed in aerobic growth with glucose as a carbon source, while *Km*ADH3 and *Km*ADH7 expression increased as cells reached stationary phase. These results are in agreement with previously published analyses of the *K. marxianus* transcriptome [21]. The *∆adh2* strain showed reduced aerobic ethanol and ethyl acetate production, suggesting a large role in ethanol and consequently ethyl acetate production. Disruption of *Km*ADH3 also had a significant effect on ethyl acetate and ethanol formation, but this is likely due to the role of *Km*ADH3 in cellular cofactor balance [45]. Under both aerobic and anaerobic conditions, the disruption of *Km*ADH2 resulted in a large accumulation of acetaldehyde, something that was not observed in the ∆*adh3* strain or the other knockouts strains, suggesting that *Km*Adh2 is the dominant enzyme in ethanol production when glucose is used as a carbon source.

With respect to *Km*ADH7, the lysate assays conducted with overexpressed *Km*Adh1–7 in *S. cerevisiae* (Fig. 7) demonstrated that *Km*Adh7 is a source of hemiacetal activity in *K. marxianus* (Fig. 2). Bioinformatic analysis revealed that *Km*Adh7 has no significant similarity to *Km*Adh1–6. NADH cofactor usage is similar to *Km*Adh1–5, but otherwise *Km*Adh7 appears to be unique among *K. marxianus* Adh enzymes. *Km*Adh7 does, however, show sequence similarity to an Adh from the bacteria *Cupriavidus necator*, but not to other yeast Adh enzymes [19]. The *C. necator* NAD(H)(P)-dependent enzyme exhibits broad substrate specificity including activity toward the oxidation ethanol and 2,3-butanediol, and the reduction of acetaldehyde, acetoin, and diacetyl; however, hemiacetal oxidation has not been demonstrated [46, 47]. Such activity has been reported for Adhs found in both *C. utilis* and *N. crassa*, as well as in *S. cerevisiae* [14–16]. The *∆adh7* strain of *K. marxianus* CBS 6556 studied here did not result in a reduction in ethyl acetate biosynthesis (Fig. 6a) and, therefore, the role of *Km*Adh7 in ester biosynthesis remains unclear.

Previous investigations of *K. marxianus* metabolism suggest that Atf activity plays a critical role in ethyl acetate biosynthesis [17, 18]. The studies presented here use new genome-editing tools and biochemical assays to confirm the function of *Km*ATF, acetate ester synthesis via the condensation of an alcohol with acetyl-CoA. CRISPR–Cas9 technology adopted for use in *K. marxianus* also allowed for the creation of a library of *Km*ADH disruption strains, identifying *Km*ADH2 as critical for the reduction of acetaldehyde to ethanol (a precursor to ethyl acetate). In addition, *Km*Adh7 was found to exhibit activity toward the oxidation of hemiacetal to ethyl acetate, but the absence of this activity in the *∆adh7* strain of CBS 6556 did not produce a measurable reduction in ethyl acetate biosynthesis. It was recently postulated that ester biosynthesis in *K. marxianus* may also occur through homologs to the medium-chain acyltransferases Eeb1 and Eht1 from *S. cerevisiae*, the isoamyl acetate-hydrolyzing esterase Iah1, the *N*-acetyltransferase Sli1 and/or the alcohol-*O-*acetyltransferase Eat1 [44, 48]. In *S. cerevisiae*, Eeb1 and Eht1 have limited activity toward the acetylation of ethanol and are most active toward medium-chain acyl-CoAs and alcohols [49]. If similar activities are found in the *K. marxianus* homologs, then these enzymes are unlikely to contribute to ethyl acetate production. Overexpression of Iah1 in *S. cerevisiae* resulted in lower ester titers due to ester hydrolysis, suggesting that the *K. marxianus* homolog may not contribute to ethyl acetate biosynthesis [50]. A recently published study describes Eat1 as a putative alcohol-*O*-acetyltransferase capable of producing ethyl acetate [48]. The new genome-editing tools created in this work should enable the future study of the role of *N*-acetyltransferases and other enzymes in ethyl acetate biosynthesis.

## Conclusion

In this work, we developed an efficient CRISPR–Cas9 system for genome editing in *K. marxianus*. This system was used to create a library of single-knockout strains to investigate ethyl acetate and ethanol biosynthetic pathways in *K. marxianus* CBS 6556. Analysis of the knockout strains revealed the importance of *Km*ADH2 in ethanol production in glucose-fed aerobic and anaerobic cultures. With respect to ethyl acetate biosynthesis, *Km*ADH2 is necessary to produce ethanol, a substrate for the Atf-catalyzed condensation reaction with acetyl-CoA. Because functional disruption of *Km*ATF did not completely abolish ethyl acetate production, alternative biosynthetic routes are likely present in *K. marxianus*. One possible pathway is the oxidation of hemiacetal (the spontaneous product of ethanol and acetaldehyde) by Adh activity, an activity that we identify in *Km*Atf7.

## Methods and materials

### Strains and culturing conditions

All strains were purchased from ATCC or DSMZ (Deutsche Sammlung von Microorganismen und Zellkulturen). All materials were purchased from Fisher Scientific unless noted otherwise. All yeast strains used in this study are listed in Additional file 1: Table S2.

Wild-type *K. marxianus* strain CBS 6556 as well as the *S. cerevisiae* BY4742 strain were grown in shake flasks containing 50 mL YM media (3 g L^−1^ yeast extract, 3 g L^−1^ malt extract, 5 g L^−1^ peptone; DB Difco^®^, Becton–Dickinson) with 10 g L^−1^ glucose. Overnight cultures were inoculated with isolated single colonies freshly grown on agar YM plates. The overnight cultures were used to inoculate shake flask at an OD of 0.05, which were subsequently cultured at 30, 37, 40, 45, and 48 °C. For *K. marxianus* cell lysate studies, strain CBS 6556 was grown for 8 h in 1 mL YM media at 37 °C. Then, 500 µL was transferred into 50 mL YM media, and culture was grown for 16 h.

To create knockout strains of *K. marxianus*, cells harboring a CRISPR–Cas9 plasmid were cultured in synthetic-defined medium without uracil (SD-U) containing 6.7 g L^−1^ yeast nitrogen base without amino acids DB Difco^®^; (Becton–Dickinson), 1.92 g L^−1^ yeast synthetic drop-out medium supplements without uracil (Sigma-Aldrich), and 20 g L^−1^ glucose or SD-U plates containing 15 g L^−1^ agar. To remove the CRISPR–Cas9 plasmid, cells were grown in YPD medium (5 g L^−1^ yeast extract, 10 g L^−1^ peptone with 20 g L^−1^ glucose; DB Difco^®^, Becton–Dickinson) overnight. Screening of XYL2 disruption colonies was achieved on SDX media containing 6.7 g L^−1^ yeast nitrogen base DB Difco^®^; (Becton–Dickinson), 0.79 g L^−1^ complete supplement mixture (CSM; Sunrise Science Products), and 20 g L^−1^ xylitol.

*K. marxianus* alcohol dehydrogenase and alcohol-*O*-acetyltransferase knockout strains (YS402, YS630, YS671, YS673, YS675, YS679, YS703, YS720, and YS794; see Additional file 1: Table S2) were cultured in uracil supplemented SD^+^U media at 37 °C (6.7 g L^−1^ yeast nitrogen base DB Difco^®^; (Becton–Dickinson), 0.79 g L^−1^ complete supplement mixture (CSM; Sunrise Science Products), and 130 mg L^−1^ uracil (Sunrise Science Products). Overnight cultures were inoculated into 25 mL of media in 250 mL baffled shake flasks (0.05 initial OD) and incubated at 37 °C for the length of the experiment. Initial and final optical cell densities (ODs) were measured using the Nanodrop 2000c UV–Vis spectrometer (Thermo Scientific) at 600 nm.

For Adh and Atf overexpression studies, *S. cerevisiae* strains (YAL1-9 and YS202, see Additional file 1: Table S2) with either an empty vector or an Adh/Atf expression plasmid were grown at 30 °C for 8 h in 1 mL SD-U and then transferred to a shake flask with 50 mL SD-U and grown for an additional 16 h.

### Bioreactor cultures

*K. marxianus* strain CBS 6556 cultures were grown in a 1-L stirred bioreactor in batch mode (Biosatat^®^A, Satorius AG). The vessel was equipped with four baffles, two Rushton impellers, gassing tube; exhaust-gas cooler; ports for supplementation, inoculation, and sampling; and sensors for dissolved oxygen, temperature, and pH. Cultures were grown in synthetic-defined (SD) media (6.7 g L^−1^ yeast nitrogen base without amino acids, DB Difco^®^; and 0.79 g L^−1^ CSM, Sunrise Science Products) containing 20 g L^−1^ glucose and 1 mL of a 1:1000 dilution of antifoaming agent (Antifoam B Emulsion, Sigma-Aldrich) at 37 °C. Twenty-five-milliliter overnight cultures were used to inoculate the reactor at an initial cell density of 0.08 OD. Dissolved oxygen (DO) concentration was maintained at 60% saturation by constant aeration with 1000 ccm air and varying stir rates. Media pH of 5 was maintained by titration with 1 M sodium hydroxide. When necessary, liquid was taken through the sampling port. Gas sampling was accomplished by collecting 0.4 L of exhaust gas in a 1-L gas-sampling bag connected to the gas-sampling port (Supel™ Inert Multi-Layer Foil Gas Sampling Bag, Sigma-Aldrich). OD was measured, and spent media and gas samples were analyzed by GC-FID. Media samples for glucose analysis were spun down to removed cells and the supernatant was stored at −20 °C prior to analysis.

### Glucose analysis

The spent media of the bioreactor experiments was analyzed for residual glucose using the Glucose (GO) Assay Kit (GAGO-20; Sigma-Aldrich). The procedure was slightly modified. Briefly, 200 µL of sample was mixed with 400 µL Glucose Assay reagent. After reaction for 30 min at 37 °C the reaction was stopped by addition of 400 µL 12 N H_2_SO_4_. 250 µL of the solution mixed was then transferred into a 96-well plate, and absorbance was determined at 540 nm using a BioTek Synergy 2 UV–Vis plate reader.

### Molecular cloning and plasmids construction

All cloning was accomplished using Phusion polymerase, restriction endonucleases, and Gibson assembly master mix purchased from New England BioLabs (NEB). DNA oligos were purchased from Integrated DNA Technologies (IDT). Chemically competent DH5α *Escherichia coli* was used for plasmid propagation. Following transformation, *E. coli* cells were grown in LB medium containing 100 mg L^−1^ ampicillin. All plasmids and primers used are listed in Additional file 1: Tables S3 and S4.

The CRISPR–Cas9 plasmid was constructed using pJSK316-GPD (a kind gift from Dr. Dae-Hyuk Kweon, Sungkyunkwan University) that contains the backbone necessary for plasmid retention in *K. marxianus* [51]. This plasmid was digested with the restriction enzymes KpnI and SacII. The Tef1p–Cas9-Cyct cassette was amplified from p414 (Addgene #43802) using primers P1379/P1525 [52]. The structural guide RNAs containing a *Sc*SNR52 promoter, an Ade2 target sequence, and the structural guide RNA (SNR52-sgRNA-Ade2; Additional file 1: Figure S7) were designed based on previously described sequences [52]. The SNR52-sgRNA-Ade2 fragment was amplified using primer P1626/P1530. The Cas9 and sgRNA fragments were inserted into the digested pJSK316-GPD by Gibson Assembly [53]. For increased editing efficiency, the Cas9 nuclease sequence was codon optimized using Optimizer (http://genomes.urv.es/OPTIMIZER/) and the codon usage table for *K. marxianus* [54]. The resulting sequence was then manually altered, as shown in Additional file 1: Figure S8, to allow for production of three similar-sized gBlocks (double stranded fragments from IDT DNA). The CRISPR plasmid (pIW333) was cut with SpeI and XhoI, and the 3 gBlocks were inserted by Gibson Assembly to create a new *Km*CRISPR plasmid (pIW360). Different RNA polymerase III promoters were designed to assess the expression of sgRNAs and CRISPR–Cas9 efficiency [27]. The xylitol dehydrogenase gene (XYL2) was used as reporter gene for Cas9-induced gene disruption. The CRISPR–Cas9 plasmids with varying sgRNA promoters were constructed by inserting different RNA polymerase III promoters into pIW360. *Sc*SNR52 was amplified from pIW360 using P1626/P1789. The *K. marxianus* RNA polymerase III promoters were amplified from isolated *K. marxianus* CBS 6556 genomic DNA. *Km*SNR52 was amplified with P1792/P1806, while P1792/P1793 were used to generate the SNR52 fragment for *Km*SNR52-tRNA^Gly^. *Km*SCR1 and *Km*RPR1 were amplified using primers P1795/P1796 and P1798/P1799, respectively. The tRNA^Gly^ sequence was amplified with primers P1839/40 for insertion by itself or with P1790/P1807, P1794/P1807, P1797/P1807, and P1800/P1807 for insertion with *Sc*SNR52, *Km*SNR52, *Km*SCR1 and *Km*RPR1, respectively. The sgRNA fragments targeting XYL2 were amplified using primers P1773/P1530 and P1775/P1530. To exchange the sgRNA target sequence, the reverse primer of the promoter fragment and the forward primer of the sgRNA fragment were replaced by appropriate primers containing the target sequence.

For ADH and ATF overexpressions in *S. cerevisiae*, the eight genes were separately cloned into the pRS426 vector containing a PGK1 expression cassette (pIW21) [13]. The ADH and ATF genes of interest were identified in the annotated genome of *K. marxianus* DMKU3-1042 as KLMA_40102, KLMA_40220, KLMA_80306, KLMA_20158, KLMA_20005 KLMA_80339, and KLMA_40624 for *Km*ADH1–7, respectively and KLMA_30203 for *Km*ATF [19]. Blast searches confirmed the presences of each gene in the unannotated genome of CBS 6556. All proteins were tagged with a C-terminal Myc tag and cloned into pIW21 at the SacII and SpeI sites using Gibson Assembly. Coding sequences for ADH2–7 and ATF were made using the primers shown in Additional file 1: Table S4 and cloned into pIW695 that was cut with SacII and AvrII. The resulting plasmids pIW696–702 are listed in Additional file 1: Table S3.

### Transformation of *K. marxianus*

Plasmid and linear DNA transformations were performed using a previously reported protocol with the following modifications [55]. In brief, 1.5 mL *K. marxianus* cells was grown to stationary phase and harvested by centrifugation at 5000 rpm for 1 min. After washing with 1 mL of sterile water, cells were suspended in 100 µg carrier DNA (salmon sperm DNA), and 0.2–1 µg of plasmid or linear DNA. 400 µL of transformation mix (40% polyethylene glycol 3350, 0.1 M lithium acetate, 10 mM Tris–HCl (pH 7.5), 1 mM EDTA, and 10 mM DTT) was then added, and the solution was incubated at room temperature for 15 min. Subsequently, the transformation mix was heat shocked at 47 °C for 15 min, and cells were plated.

### Creation of a URA3 auxotrophic strain

To create a URA3 disruption strain, a truncated *K. marxianus* URA3 fragment (missing 160 bp of the coding region) with 500 bp homology upstream and downstream was transformed into *K. marxianus* strain CBS 6556. Transformed cells were recovered overnight and plated on 5-fluororotic acid (5-FOA) containing plates. Solid media contained 6.7 g L^−1^ yeast nitrogen base (Becton–Dickinson), 1.92 g L^−1^ yeast synthetic drop-out medium supplements without uracil (Sigma-Aldrich), 50 mg L^−1^ uracil (Sunrise Science Products), 1 g L^−1^ 5-FOA (Sigma-Aldrich) and 20 g L^−1^ glucose. Colonies were selected based on colony PCR and selected colonies were sequenced (Additional file 1: Figure S9). To create the URA3 disruption homology donor, overlap extension PCR was performed as previously described [56]. Overlapping fragments of upstream and downstream regions of the sequence targeted for deletion are amplified using primers P1019/P1920 and P1021/P1022, respectively. Resulting fragments are purified and used for overlap PCR with P1019/P1022. The resulting fragment was purified and used for transformation. To confirm disruption, genomic DNA was screened using primers P1072/1073 where a knockout resulted in a 200 bp amplified fragment, with the wild-type gene producing a 360 bp fragment. The knockout design is shown in Additional file 1: Figure S10.

### CRISPR–Cas9-mediated gene disruption

The *K. marxianus* CRISPR–Cas9 system developed in this work was adopted from systems developed for *Y. lipolytica* and *S. cerevisiae* [27, 52]. Cas9 was codon optimized for *K. marxianus* and was expressed from a plasmid using the *S. cerevisiae* Tef1 promoter. For sgRNA expression, RNA polymerase III promoters in *K. marxianus* were identified by blasting the *K. lactis* genes of SNR52 (NC_006042.1), RPR1 (NC_006042.1), and SCR1 (NC_006042.1) against the draft genome of *K. marxianus* strain CBS 6556 (Accession Number: AKFM00000000) [57]. The search yielded *K. marxianus* SNR52, RPR1 and SCR1 that had 86, 75, and 82% identity to the respective *K. lactis* genes. The promoter regions were identified by searching for conserved A and B-box motifs as previously described [27, 58]. Promoter regions were defined as ~100 bp upstream of the A box until the start of the coding region of the gene. For *Km*SCR1, the boxes were within the transcribed region, which is why the promoter was chosen as the start of the aligned sequence to about 30 bp downstream of the identified B-box. The glycine tRNA (tRNA^Gly^) was identified by blasting the annotated tRNA-Gly (AGG) from *K. marxianus* strain DMKU3-1042 (RNA central; URS00003CECDB; [19, 50]) against the genome of CBS 6556 as described above.

sgRNA target sequences for xylose dehydrogenase (XYL2), ADH and ATF knockouts were identified using the sgRNA design tool hosted by the Broad Institute (http://www.broadinstitute.org/rnai/public/analysis-tools/sgrna-design) [30]. Target sequences were checked for secondary structures using the IDT OligoAnalyzer Tool 3.1 (https://www.idtdna.com/calc/analyzer) and uniqueness within the *K. marxianus* genome using BLAST. All sgRNA sequences are listed in Table 2 and Additional file 1: Table S1.

For ADH and ATF disruptions, transformed *K. marxianus* cells were cultured in 2 mL SD-U media or plated on SD-U plates and grown for 2 days at 30 °C. If cultured in liquid media, 50 µL of cell culture was transferred into new media after 1 day of culturing. After 2 days of growth colonies were screened by amplifying the CRISPR–Cas9-edited region in the genome by colony PCR and subsequent sequencing of the purified PCR fragments. Positively confirmed disruptions colonies were saved at −80 °C after the plasmid was removed.

### Adh and Atf protein sequence analysis

Homology of the *K. marxianus* Adh1–7 and Atf proteins to other proteins was analyzed by Pairwise Sequence Alignment using the EMBOSS Needle software form EMBL-EBI. Analyzed sequences are shown in Additional file 1: Table S5.

### Headspace gas chromatography

One-milliliter of culture supernatant or cell lysate reaction was used for headspace GC analysis in a 10 mL headspace vial containing 1 g of NaCl and 20 µL of 5 g L^−1^ 1-pentanol as internal standard. Volatile metabolite concentration was measured using an Agilent 7890A system equipped with an Agilent DB-624UI column and an FID detector. For metabolite separation, the temperature was held at 40 °C for 2 min, then increased 20 °C min^−1^ to 70 °C and 50 °C min^−1^ to 220 °C and held for 2 min. For the bioreactor off gas analysis 1 mL of the off gas was injected from the gas sample bag by manual injection.

### Reverse transcription quantitative PCR (RT-qPCR)

Total RNA was extracted using the YeaStar™ RNA Kit (Zymo Research). RNA was DNAse treated (DNAse I, NewEngland Biolabs) for 30 min. and subsequently purified using the RNA Clean & Concentrator™-5 Kit (Zymo Research). RNA was used for the reverses transcription reaction (iScript™ Reverse Transcription Supermix for RT-qPCR, Bio-Rad) and cDNA was used for SYBR Green qPCR (SsoAdvanced™ Universal SYBR^®^ Green Supermix. Bio-Rad) using the Bio-Rad CFX Connect™. Primers used for the qPCR reaction are listed in Additional file 1: Table S4. For Figs. 4a and 5a, b (top panel) total copy number was calculated from a standard curve with GAPDH as an internal standard. Fold changes in Fig. 5a, b (bottom panel) were calculated under consideration of the reaction efficiency as previously described [59]. Log and stationary phase expression were compared with lag phase expression using transcript level normalized to GAPDH.

### Total cell lysates

Cell lysis was performed as described earlier [13]. In short, cell were harvested, washed, and resuspended in equal volumes of wet cell pellets, 425–600 μm acid-washed glass beads (G8772, Sigma-Aldrich), and ice-cold lysis buffer (100 mM potassium phosphate buffer, 2 mM magnesium chloride, 5 mM DTT, and Pierce™ Protease Inhibitor Tablets). The cells were disrupted at 4 °C by vortexing 10 times for 30 s with a 30 s cooling step between each vortexing. The beads were removed by centrifugation at 500*g* for 5 min at 4 °C, and the supernatant transferred to a pre-cooled 1.5 mL tube. The protein concentrations of whole cell lysates were determined by Pierce™ 660 nm Protein Assay.

### Enzyme activity assay

*K. marxianus* strain CBS 6556 and *S. cerevisiae* strains containing Adh and Atf expression plasmids are described in Additional file 1: Table S1. One-hundred micrograms of total cell lysate isolated from the various strains was used in each reaction. Lysates were incubated in 100 mM potassium phosphate (pH 7.4), 500 mM ethanol, and 0.5 mM acetyl-CoA to test for Atf activity and 100 mM potassium phosphate (pH 7.4), 100 mM acetaldehyde, 1 M ethanol, and 30 mM NAD^+^ (Sigma-Aldrich) to test for hemiacetal activity of Adh. To test for esterase activity, cell lysates were incubated in potassium phosphate (pH 7.4), 500 mM ethanol, and 500 mM potassium acetate. The reaction mixtures were incubated for 30 min at 30 °C. The samples were analysis by headspace chromatography as described above. To allow for hemiacetal production, reaction mixtures with 10 M ethanol and 1 M acetaldehyde were mixed beforehand, and pH was adjusted to 10 using sodium hydroxide as previously described [15]. The chromatograms of the hemiacetal solution and vector control experiments showed no significant peak for ethyl acetate (Additional file 1: Figure S11).

### Western blot analysis

Western blot analysis was performed to confirm the expression of Adh and Atf proteins with C-terminal c-Myc tag. 2.5 OD of cells were lysed using 0.1 M NaOH. Samples were loaded onto a 10-well Any kD™ Mini-PROTEAN^®^ TGX™ Precast Protein Gel (BioRad) and run for 1 h at 150 V. Samples were then electrophoretically transferred overnight to a PVDF membrane at 25 V. Membranes were blocked with 5% nonfat milk in TBST buffer for 1 h at room temperature and incubated with anti-c-Myc mouse antibody (Sc-40, Santa Cruz Biotech) or anti-GAPDH (PA1-987) diluted to 1:2000 and 1:5000 in TBST buffer with 1% nonfat milk. Goat anti-mouse IgG-HRP (31,430) diluted to 1:10,000 was added as secondary antibody and incubated at room temperature for 30 min. After washing with TBST, HRP substrate (Bio-Rad) was used for signal detection. Blots were imaged using the BioRad ChemiDoc™ MP System with the Image Lab software.

### Statistical analysis

Data points represent arithmetic means of at least triplicate biological samples, and error bars represent the standard deviation. Comparisons between two samples were accomplished by an unpaired two-tailed *T* test with a significant difference at *P* < 0.05. Groups of samples were analyzed by one-way ANOVA with a Tukey post hoc test and considered significant at *P* < 0.05. Statistical analysis and plotting of data points were performed using the GraphPad Prism software.

## Additional file

**Additional file 1.** Additional figures and tables.

## Abbreviations

CRISPR: clustered regularly interspaced short palindromic repeats
Adh: alcohol dehydrogenase
Atf: alcohol-*O*-acetyltransferase
Acetyl-CoA: acetyl coenzyme A
NAD(P)(H): nicotinamide adenine dinucleotide (phosphate) (reduced)
sgRNA: single guide RNA
RT-qPCR: reverse transcription quantitative polymerase chain reaction
5-FOA: 5-fluororotic acid
OD: optical density
DO: dissolved oxygen
EDTA: ethylenediaminetetraacetic acid
DTT: dithiothreitol
TBST: tris-buffered saline Tween 20
HRP: horseradish peroxidase
