# Supplementary material for: CRISPR–Cas9-enabled genetic disruptions for understanding ethanol and ethyl acetate biosynthesis in Kluyveromyces marxianus
Source: Biotechnol Biofuels. 2017 Jun 24;10:164. doi: 10.1186/s13068-017-0854-5 (PMC5483312; doi:10.1186/s13068-017-0854-5)
Supplement: Supplementary file 1 — Additional file 1. Additional figures and tables. [file 13068_2017_854_MOESM1_ESM.docx]

**Additional Files**

Research Article

**CRISPR–Cas9-enabled genetic disruptions for understanding ethanol and ethyl acetate biosynthesis in *Kluyveromyces marxianus***

Ann-Kathrin Löbs^1^

Ronja Engel^1,2^

Cory Schwartz^1^

Andrew Flores^1^

Ian Wheeldon^1^

^1^Department of Chemical and Environmental Engineering, UC Riverside, Riverside, USA

^2^ Mannheim University of Applied Sciences, Mannheim, Germany

**Correspondence:** Dr. Ian Wheeldon, Chemical and Environmental Engineering, University of California, Riverside, 900 University Ave, 92521, Riverside, USA

**E-mail**: iwheeldon@engr.ucr.edu


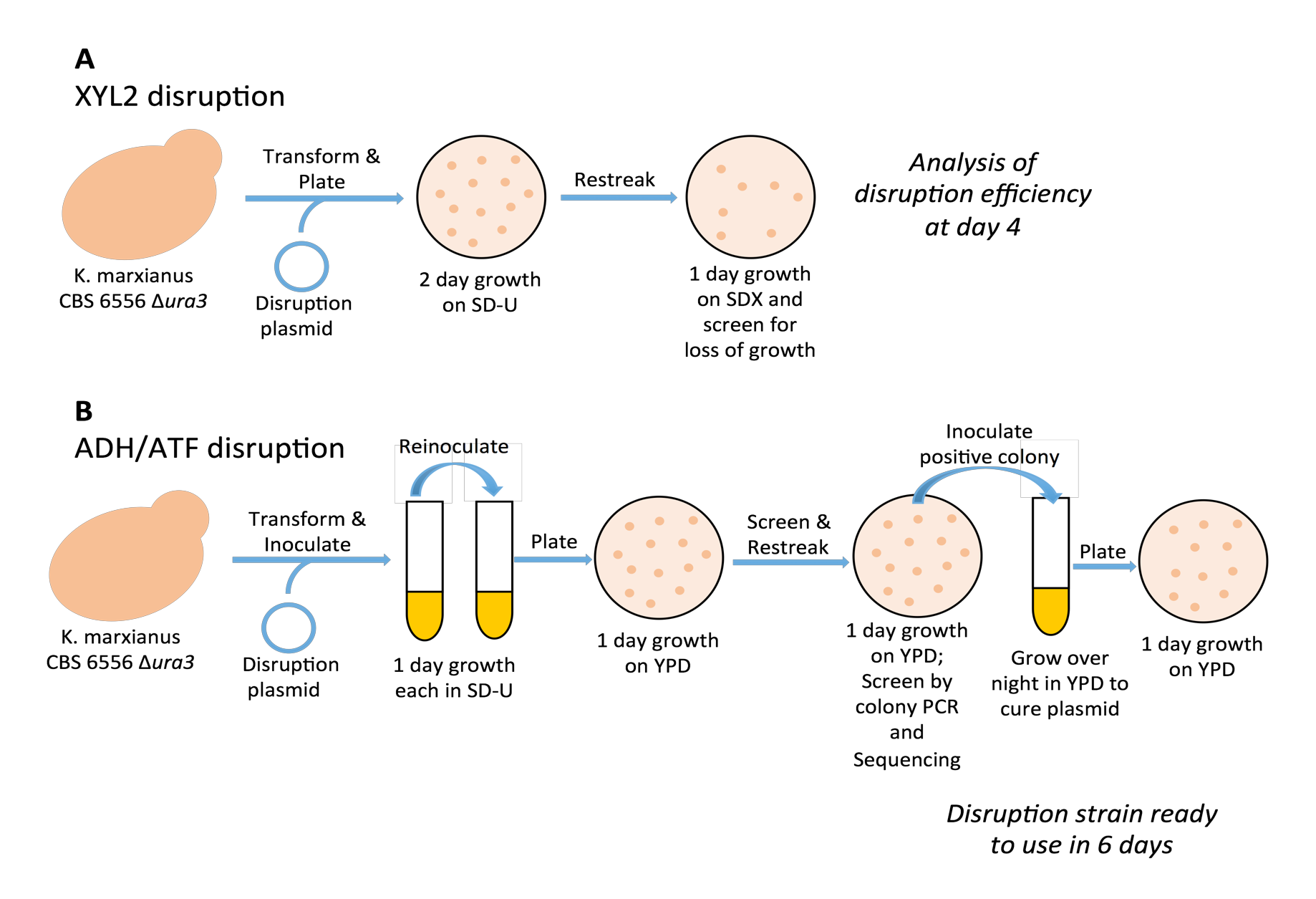


**Figure S1: Schematic workflow of CRISPR-Cas9 mediated gene disruption and screening.** Description of the workflow for XYL2 (A) and ADH/ATF (B) disruptions and screening. (A) To determine the efficiency of the sgRNA expression promoter systems, disruptions were screened by the phenotypic loss of growth on xylitol. After transformation, cells were plated on selective media and grown for 2 days. Colonies were restreaked on rich (YPD) and xylitol (SDX) solid media. At day 4, colonies with growth on rich but no growth on SDX media were considered to have a disruption in the XYL2 gene. Selected colonies were confirmed by sequencing. (B) For ADH and ATF disruptions, cells were transformed and grown in selective media for 1 day. To enrich colonies with the plasmid, cells were reinoculated in new media for another day and subsequently plated on rich media. Random colonies selected for screening were subjected to colony PCR and restreaked on fresh solid media plates. Colony PCR products were sequenced to identify indels. Finally, colonies with successful gene disruptions were grown over night in rich media to cure the CRIPSR-Cas9 plasmid.

| **Target** | **Target Sequence** | **Score** | **Strand** | **Indel Success** | **sgRNA promoter** |
| --- | --- | --- | --- | --- | --- |
| XYL2 T1 | ACGATCGCCAACCTTGACCA | 0.66 | antisense | 68/90 | **ScSNR52-tRNA^Gly^** |
| ADH1 | AAAGAACGTCGACTTGGCCG | 0.832 | sense | 0/20 |  |
| ADH1 T2 | GGCAGCCTGGACAGCGTCAG | 0.486 | antisense | 0/20 |  |
| ADH1T3 | AGACTTCAAAGCCTTGTACA | 0.453 | antisense | 2/10 |  |
| ADH2 | GTGACCTTGCCGGTATCAAA | 0.686 | sense | 0/20 |  |
| ADH2 T2 | GTCACCAGCCTTCATTTCAG | 0.697 | antisense | 0/20 |  |
| ADH2 T3 | GGTACCAGCTGGGATGTGAG | 0.421 | antisense | 4/30 |  |
| ADH3 | GCTATTCCAGAAAAGCAAAA | 0.826 | sense | 2/10 |  |
| ADH4 | GCCATCCCAGAATCCCAAAA | 0.825 | sense | 4/10 |  |
| ADH5 | ATGGTCTTGAAAGAACACAA | 0.716 | sense | 1/10 |  |
| ADH6 | GTACCACCACCGCAAAGTAG | 0.751 | antisense | 2/10 |  |
| ADH7 | GTATTAGGCCATGAAGGTAT | 0.709 | sense | 0/20 |  |
| ADH7 T2 | TCTCCTTAGCCATAGCCAAA | 0.883 | antisense | 0/20 |  |
| ADH7 T3 | GCTTGAGCTGAGAGATTGAT | 0.695 | antisense | 3/5 |  |
| ATF T1 | GCTGAAACAGAGTTTCAGCA | 0.958 | sense | 0/20 |  |
| ATF T2 | ATATAGTCTTCGGCAACACC | 0.551 | antisense | 4/10 |  |
| XYL2 T1 | ACGATCGCCAACCTTGACCA | 0.622 | antisense | 58/90 | **KmRPR1-tRNA^Gly^** |
| XYL2 T2 | GCAATTCAAGATAAGTTGGG | 0.839 | sense | 2/30 |  |
| scrambled | AGTCCGGTCATTACAACTTA | - | - | 0 |  |

**Table S1: sgRNA and efficiencies used in the study**

**
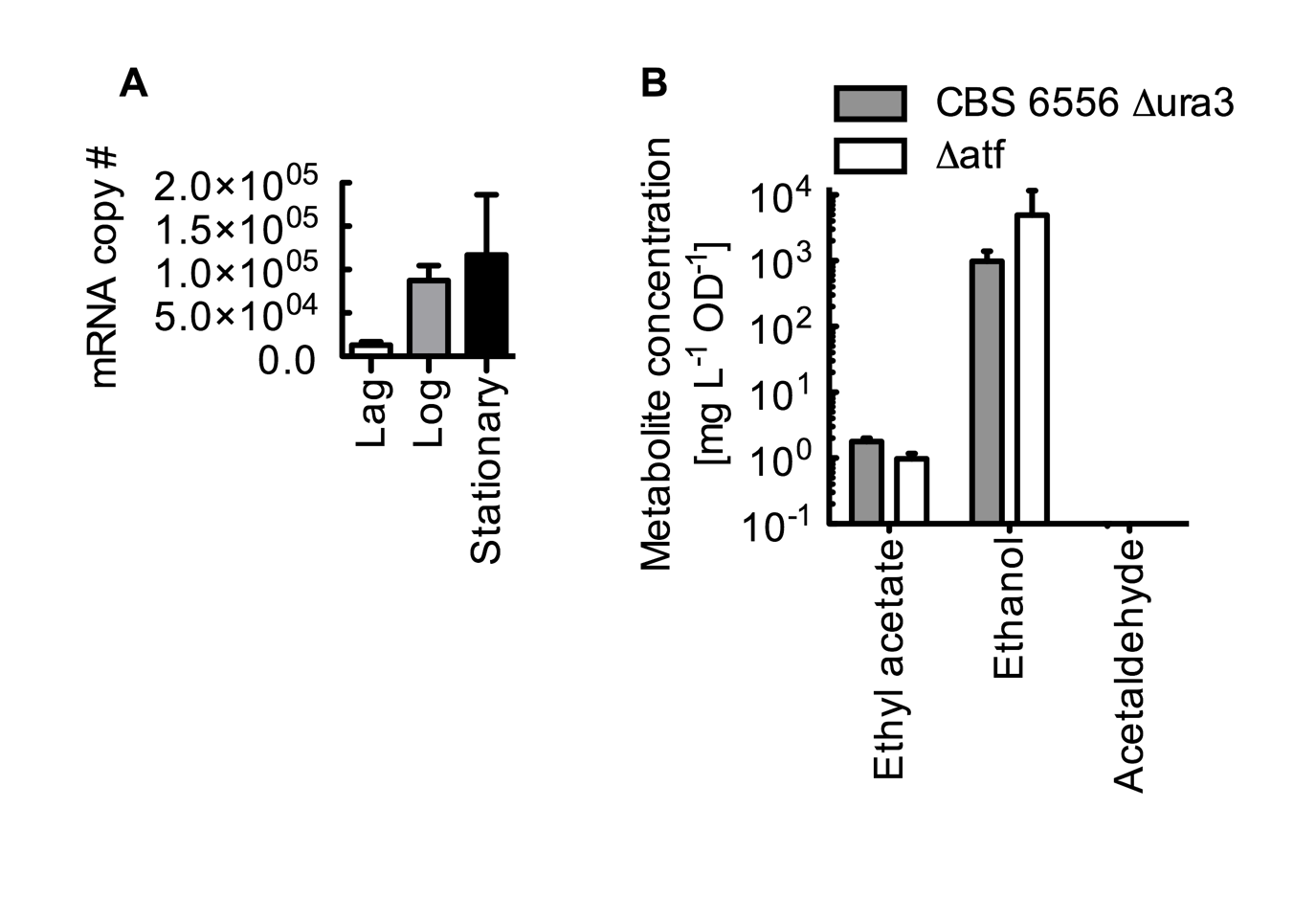
**

**Figure S2: Anaerobic ATF expression and impact of *Km*ATF knockout on volatile metabolite production.** (A) mRNA copy number of 5 ng input total RNA of wildtype *Km*ATF at different growth stages of anaerobic growth and (B) ethyl acetate, ethanol and acetaldehyde production of the URA3 deficient background strain compared to the *Km*ATF disruption strain. Bars and error bars represent the arithmetic mean and standard deviation of three samples.

**
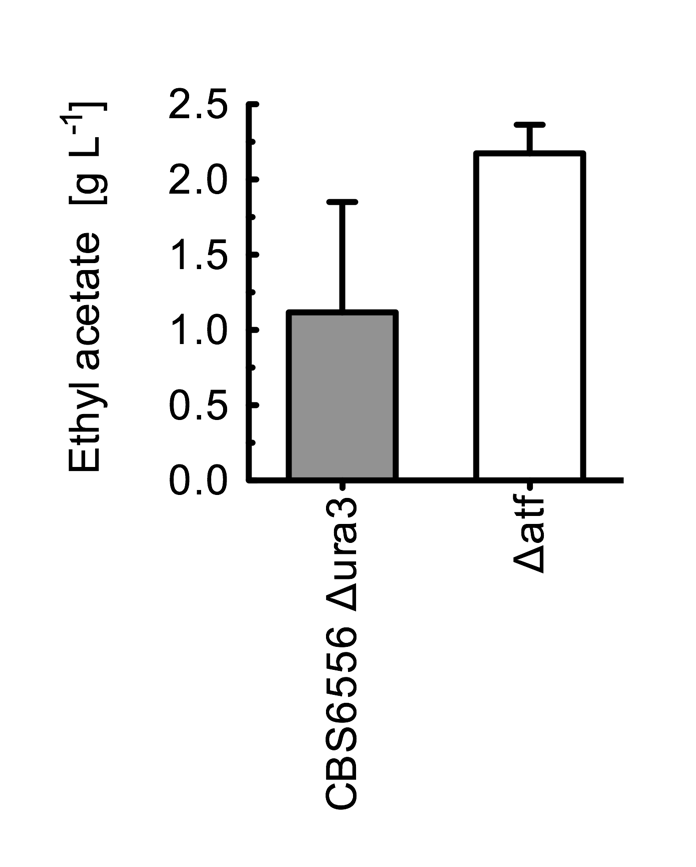
**

**Figure S3: Aerobic ethyl acetate production of *Km*ATF knockout on SD media.** *Km*ATF disruption does not lead to a significant decrease in ethyl acetate production compared to the URA3 deficient strains when the strains are grown aerobically on SD media. Bars and error bars represent the arithmetic mean and standard deviation of three samples.

**
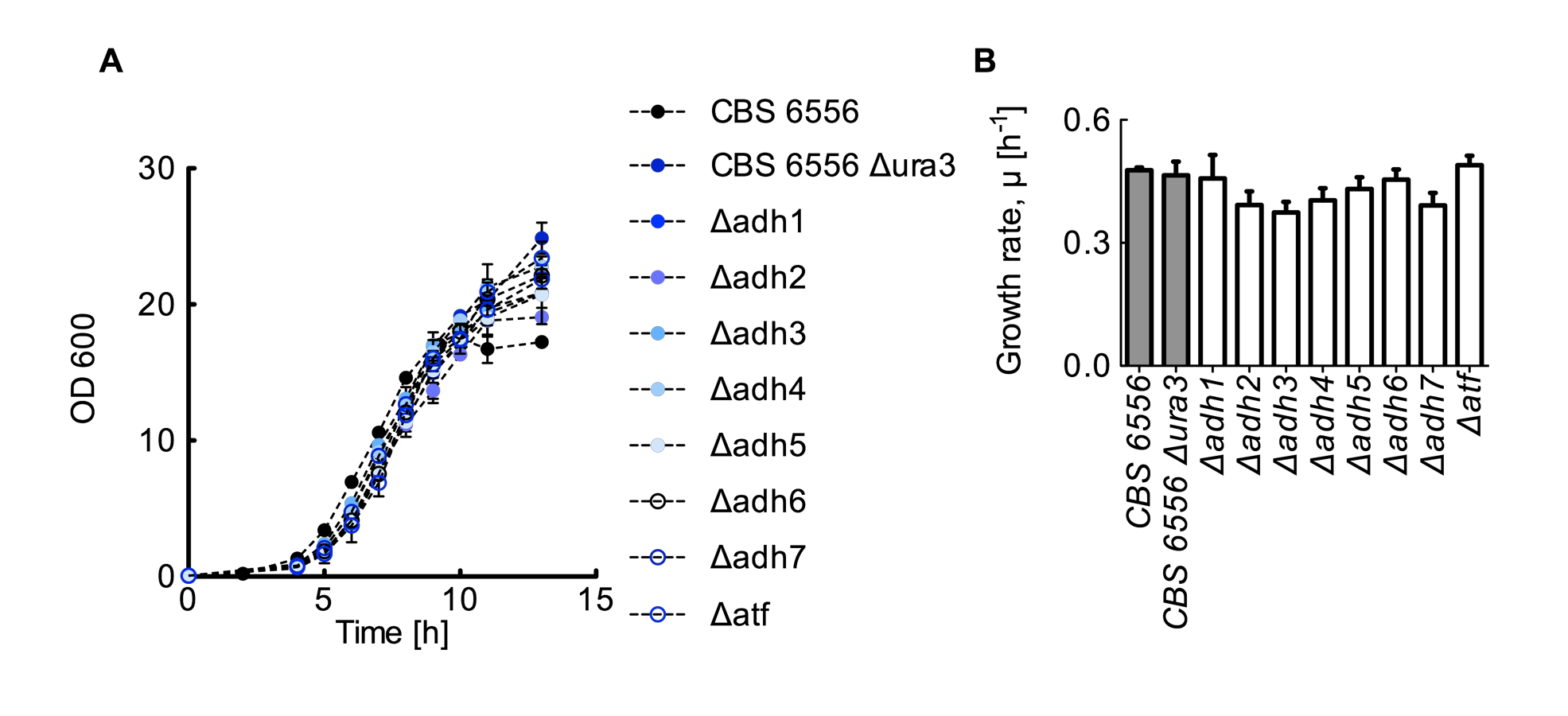
**

**Figure S4: Aerobic growth of *Km*ADH and *Km*ATF disruption strains.** (A) Growth curves and (B) growth rates of *K. marxianus* CBS 6556 wild type, URA3 deficient and *KmADH1-7* and *Km*ATF disruption strains under aerobic conditions. Data points and bars represent the arithmetic mean of 3 biological replicates and error bars represent the standard deviation.


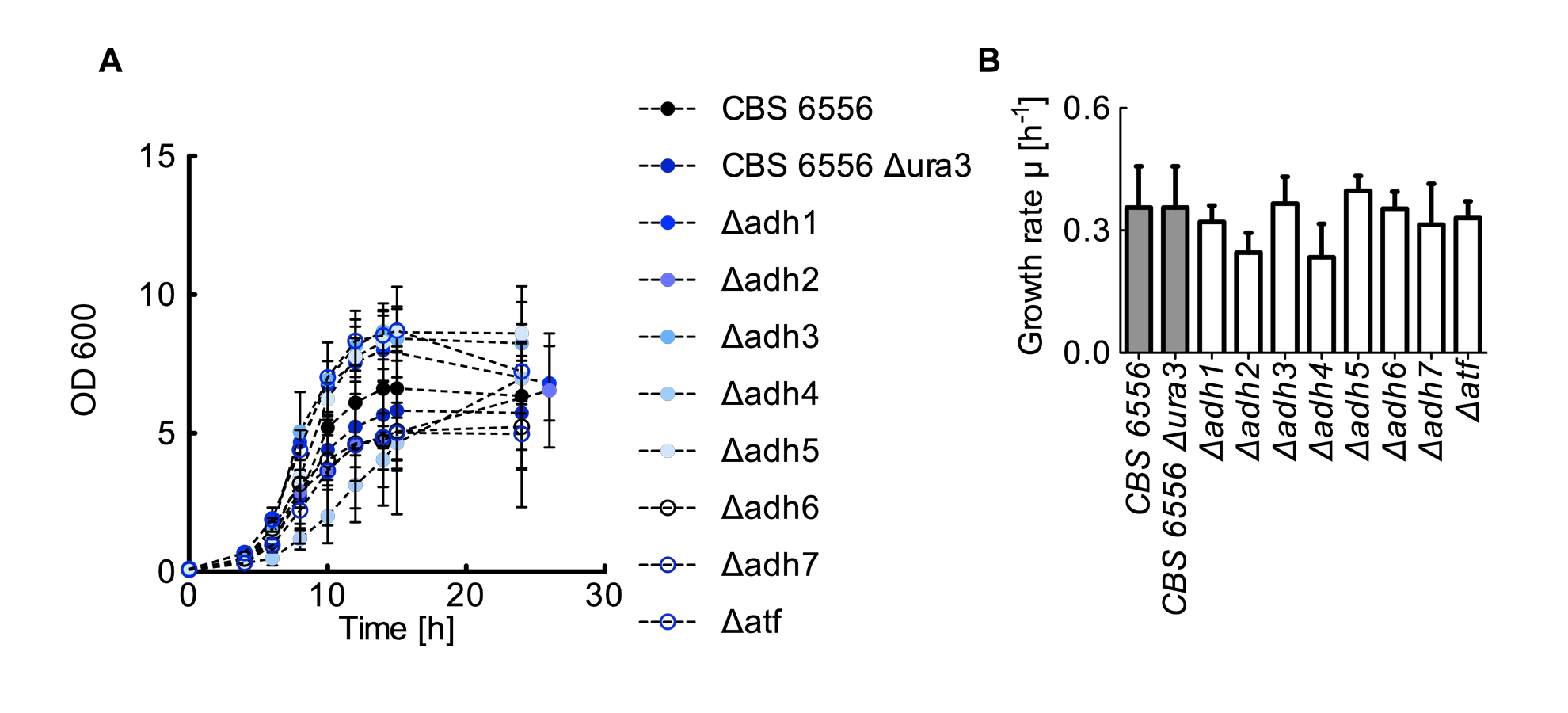


**Figure S5: Anaerobic growth of *Km*ADH and *Km*ATF disruption strains.** (A) Growth curves and (B) growth rates of *K. marxianus* wild type CBS 6556, URA3 deficient and *Km*ADH1-7 and *Km*ATF disruption strains under anaerobic conditions. Data points and bars represent the arithmetic mean of triplicate biological replicates and error bars represent the standard deviation.

**
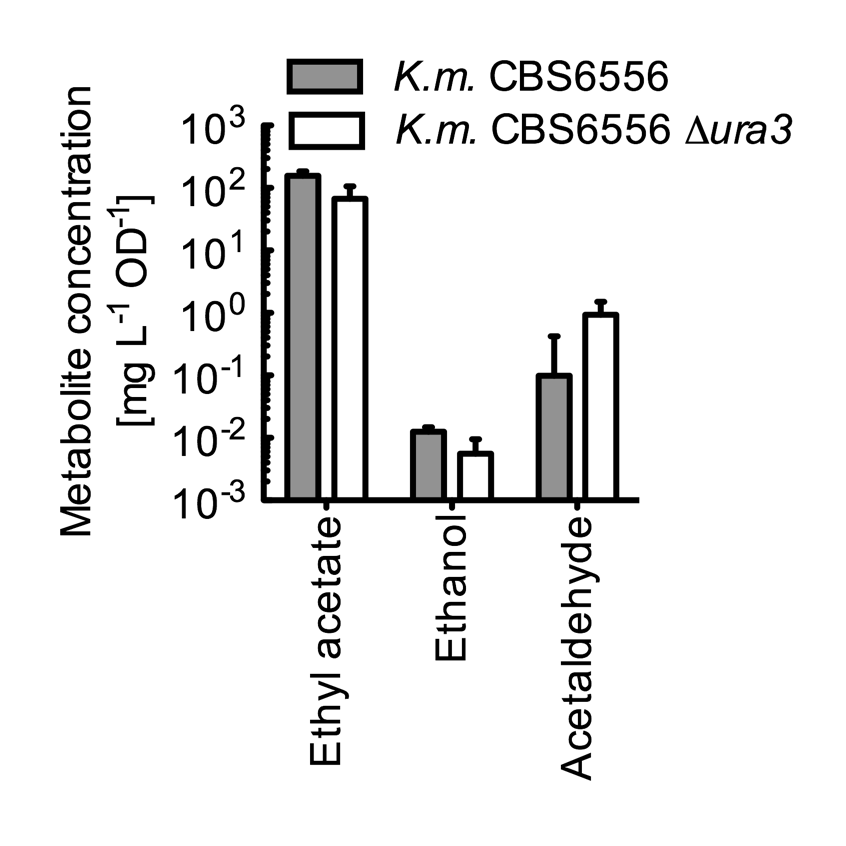
**

**Figure S6: Metabolite production of wild type and URA3 deficient *K. marxianus* CBS6556 strains.** Ethyl acetate and ethanol production are significantly reduced in the URA3 disrupted strain. Bars represent the arithmetic mean of biological triplicates and error bars represent the standard deviation.

**Table S2: Strains used in the study**

| **Strain** | **Genotype** | **Source** |
| --- | --- | --- |
| YS5 | *S. cerevisiae* BY4742 MAT*α his3Δ1 leu2Δ0 lys2Δ0 ura3Δ0* | GE Healthcare |
| YS8 | *S .cerevisiae* YS5 + pIW14 | Lin et al [1] |
| YS202 | *S .cerevisiae* YS5 + pIW107 | Zhu *et al.*[2] |
| YS302 | *K. marxianus* CBS6556 | DMSZ |
| YS402 | *K. marxianus* CBS6556 *Δura3* | This study |
| YS630 | *K. marxianus* CBS6556 *Δura3 Δadh4* | This study |
| YS671 | *K. marxianus* CBS6556 *Δura3 Δadh3* | This study |
| YS673 | *K. marxianus* CBS6556 *Δura3 Δadh5* | This study |
| YS675 | *K. marxianus* CBS6556 *Δura3 Δadh6* | This study |
| YS679 | *K. marxianu*s CBS6556 *Δura3 Δatf* | This study |
| YS703 | *K. marxianus* CBS6556 *Δura3 Δadh2* | This study |
| YS720 | *K. marxianus* CBS6556 *Δura3 Δadh7* | This study |
| YS794 | *K. marxianus* CBS6556 *Δura3 Δadh1* | This study |
| YAL1 | *S .cerevisiae* YS5 + pIW695 | This study |
| YAL2 | *S .cerevisiae* YS5 + pIW696 | This study |
| YAL3 | *S .cerevisiae* YS5 + pIW697 | This study |
| YAL4 | *S .cerevisiae* YS5 + pIW698 | This study |
| YAL5 | *S .cerevisiae* YS5 + pIW699 | This study |
| YAL6 | *S .cerevisiae* YS5 + pIW700 | This study |
| YAL7 | *S .cerevisiae* YS5 + pIW701 | This study |
| YAL8 | *S .cerevisiae* YS5 + pIW702 | This study |

**Table S3: Plasmids used in the study**

| **Name** | **Description** | **Source** |
| --- | --- | --- |
| pIW14 | pRS426 PGK1p-PGK1t | Lin et al [1] |
| pIW21 | pRS426 PGK1p-ATF1-GFP-PGK1t | Lin et al [1] |
| pIW107 | pRS426 PGK1p-ATF1-C-Myc-PGK1t | Zhu *et al*. [2] |
| pIW243 | p414-TEF1p-Cas9-CYC1t (S.c. expression, *S. pyogenes* human optimized) | Addgene [3] |
| pIW272 | pJSKM316GPD | Lee *et al*.[4] |
| pIW333 | Tef1-Cas9-Cyc1 ScSNR52-Ade Target2-SUP4 | This study |
| pIW360 | Tef1-KmCas9-Cyc1 ScSNR52-Ade Target2-SUP4 | This study |
| pIW443 | Tef1-KmCas9-SV40-Cyc1 ScSNR52-XDH1-SUP4 | This study |
| pIW444 | Tef1-KmCas9-SV40-Cyc1 KmSNR52-XDH1-SUP4 | This study |
| pIW445 | Tef1-KmCas9-SV40-Cyc1 KmSNR52p-Gly-tRNA-XDH1-SUP4 | This study |
| pIW446 | Tef1-KmCas9-SV40-Cyc1 KmSCR1p-Gly-tRNA-XDH1-SUP4 | This study |
| pIW447 | Tef1-KmCas9-SV40-Cyc1 KmRPR1p-Gly-tRNA-XDH1-SUP4 | This study |
| pIW461 | Tef1-KmCas9-SV40-Cyc1 ScSNR52-Gly-tRNA-ADH1-SUP4 | This study |
| pIW462 | Tef1-KmCas9-SV40-Cyc1 ScSNR52-Gly-tRNA-ADH2-SUP4 | This study |
| pIW463 | Tef1-KmCas9-SV40-Cyc1 ScSNR52-Gly-tRNA-ADH3-SUP4 | This study |
| pIW464 | Tef1-KmCas9-SV40-Cyc1 ScSNR52-Gly-tRNA-ADH4-SUP4 | This study |
| pIW465 | Tef1-KmCas9-SV40-Cyc1 ScSNR52-Gly-tRNA-ADH5-SUP4 | This study |
| pIW466 | Tef1-KmCas9P-SV40-Cyc1 ScSNR52-Gly-tRNA-ADH6-SUP4 | This study |
| pIW467 | Tef1-KmCas9-SV40-Cyc1 ScSNR52-Gly-tRNA-ADH7-SUP4 | This study |
| pIW468 | Tef1-KmCas9-SV40-Cyc1 ScSNR52-Gly-tRNA-ATF-SUP4 | This study |
| pIW492 | Tef1-KmCas9-SV40-Cyc1 KmRPR1-Gly-tRNA-Xdh2-SUP4 | This study |
| pIW502 | Tef1-GFP-SV40-Cyc1 Gly-tRNA-XDH1-SUP4 | This study |
| pIW503 | Tef1-GFP-SV40-Cyc1 KmRPR1-Gly-tRNA-scramble DNA-SUP4 | This study |
| pIW506 | Tef1-KmCas9-SV40-Cyc1 ScSNR52-Gly-tRNA-ATF T2-SUP4 | This study |
| pIW508 | Tef1-KmCas9-SV40-Cyc1 ScSNR52-Gly-tRNA-ADH1T2-SUP4 | This study |
| pIW509 | Tef1-KmCas9-SV40-Cyc1 ScSNR52-Gly-tRNA-ADH2 T2-SUP4 | This study |
| pIW554 | Tef1-KmCas9-SV40-Cyc1 ScSNR52-Gly-tRNA-ADH7 T2-SUP4 | This study |
| pIW557 | Tef1-KmCas9-SV40-Cyc1 ScSNR52-Gly-tRNA-ADH1 T3-SUP4 | This study |
| pIW558 | Tef1-KmCas9-SV40-Cyc1 ScSNR52-Gly-tRNA-ADH2 T3-SUP4 | This study |
| pIW576 | Tef1-KmCas9-SV40-Cyc1 ScSNR52-Gly-tRNA-ADH7 T3-SUP4 | This study |
| pIW695 | PGK1p-KmAdh1-c-Myc-PGK1t | This study |
| pIW696 | PGK1p-KmAdh2-c-Myc-PGK1t | This study |
| pIW697 | PGK1p-KmAdh3-c-Myc-PGK1t | This study |
| pIW698 | PGK1p-KmAdh4-c-Myc-PGK1t | This study |
| pIW699 | PGK1p-KmAdh5-c-Myc-PGK1t | This study |
| pIW700 | PGK1p-KmAdh6-c-Myc-PGK1t | This study |
| pIW701 | PGK1p-KmAdh7-c-Myc-PGK1t | This study |
| pIW702 | PGK1p-KmAtf-c-Myc-PGK1t | This study |

**Table S4: Primers used in the study**

| **Name** | **Primer Sequence (5' to 3')** |
| --- | --- |
| P1019 URA 1F Overl | GAGCATCTTGGTCTTCTGAG |
| P1020 URA 2R Overl | GCCCGCACCAGTCACACCGTGTGCATTGGTTACATGTGTCTTCAATAGACAGATA |
| P1021 URA 3F Overl | CCATATATCTGTCTATTGAAGACACATGTAACCAATGCACACGGTGTGACTGGTGCGG |
| P1022 URA 4R Overl | GTATACAATGTGACGCAATGC |
| P1072 URA3- F | GTC AAA CTT ATG TGC TTC TCT TG |
| P1073 URA3- R | CGGCAAGCATTAACAACCC |
| P1379 Cas9 F | CGACTCACTATAGGGCGAATTGGAGCTCCACATAGCTTCAAAATGTTTCTACTCCTTTTT |
| P1525 Cas9 R | TTATCTTTTCAAAGAcgcGGTACCaagGCAAATTAAAGCCTTCGAGCGTCCCAAAAC |
| P1530 sgRNA R | ATTAACCCTCACTAAAGGGAACAAAAGCTGctaCCGCGGcagAGACATAAAAAACAAAAAAAGCACC |
| P1626 SNR52 F | CGCTCGAAGGCTTTAATTTGCcttCCTAGGGCGTCTTTGAAAAGATAATG |
| P1684 Adh1 KO Seq F | GCCATTGGTGGGTGGTCACG |
| P1685 Adh1 KO Seq R | TATCCTTGGTCTTGGTAAAGTC |
| P1690 Adh4 KO Seq F | TTACCCGGACTTTATCGAATTAG |
| P1691 Adh4 KO Seq R | TAGTCACCGATCTCGAAGTT |
| P1692 Adh5 KO Seq F | TCCACAGTTGGTTTTTACATG |
| P1693 Adh5 KO Seq R | ATATGGTTGTCAATAATAGCCATC |
| P1694 Adh6 KO Seq F | ACCACTGGTATCAAGGTTGG |
| P1695 Adh6 KO Seq R | ACGATCAAGTCCAATTTGTCA |
| P1698 Atf KO Seq F | ATGATGAAACTACAGAAATTGTCG |
| P1699 Atf KO Seq R | AGGAAGCAGGATCAGTCTGA |
| P1773 XDH1 sgRNA F | ACGATCGCCAACCTTGACCATTTTAGAGCTAGAAATAGCAAGTTAAA |
| P1775 XDH2 sgRNA F | GCAATTCAAGATAAGTTGGGTTTTAGAGCTAGAAATAGCAAGTTAAA |
| P1789 ScSNR52 tRNA R | GAAATTGCGGCCGCGATCATTTATCTTTCACTGCGG |
| P1790 tRNA ScSNR52 F | AATGATcgcggccgCAATTTCTCTTCTACCACGAACTC |
| P1792 KmSNR52 F | CGCTCGAAGGCTTTAATTTGCcttCCTAGGgcgGGGAGTGAGTAAAAAAAAGAGAAG |
| P1793 KmSNR52 tRNA R | AGAAATTGcggccgcGTAAGATTCGAACTGCGGACG |
| P1794 tRNA KmSNR52 F | ATCTTAcgcggccgCAATTTCTCTTCTACCACGAACTC |
| P1795 KmSCR1 F | CGCTCGAAGGCTTTAATTTGCcttCCTAGGgcgTGTTATACTTGGATAAGTGGCTC |
| P1796 KmSCR1 tRNA R | agaaattgcggccgcTGGGAAAATTTGCTAAATCGTTAC |
| P1797 tRNA SCR1 F | tttcccagcggccgCAATTTCTCTTCTACCACGAACTC |
| P1798 KmRPR1 F | CGCTCGAAGGCTTTAATTTGCcttCCTAGGgcgTATACTCCAACTTGGTCGAAAG |
| P1799 KmRPR1 tRNA R | agaaattgcggccgcATCTAAATTCTCTCTTTTTCCTTCAA |
| P1800 tRNA RPR1 F | tttagatgcggccgCAATTTCTCTTCTACCACGAACTC |
| P1806 KmSNR52 Xdh1 R | ATGGTCAAGGTTGGCGATCGTCctcgagGGTAAGATTCGAACTGCGGACG |
| P1807 GlytRNA Xdh1 R | AAAATGGTCAAGGTTGGCGATCGTCctcgagGTTGACACTGACGGGATTCGA |
| P1811 ADH1 KO sgRNA F | AAAGAACGTCGACTTGGCCGTTTTAGAGCTAGAAATAGCAAGTTAAA |
| P1812 ADH1 KO R | CGGCCAAGTCGACGTTCTTTCctcgagGTTGACACTGACGGGATTC |
| P1813 ADH2 KO sgRNA F | GTGACCTTGCCGGTATCAAATTTTAGAGCTAGAAATAGCAAGTTAAA |
| P1814 ADH2 KO R | TTTGATACCGGCAAGGTCACCctcgagGTTGACACTGACGGGATTC |
| P1815 ADH3 KO sgRNA F | GCTATTCCAGAAAAGCAAAATTTTAGAGCTAGAAATAGCAAGTTAAA |
| P1816 ADH3 KO R | TTTTGCTTTTCTGGAATAGCCctcgagGTTGACACTGACGGGATTC |
| P1817 ADH4 KO sgRNA F | GCCATCCCAGAATCCCAAAATTTTAGAGCTAGAAATAGCAAGTTAAA |
| P1818 ADH4 KO R | TTTTGGGATTCTGGGATGGCCctcgagGTTGACACTGACGGGATTC |
| P1819 ADH5 KO sgRNA F | ATGGTCTTGAAAGAACACAATTTTAGAGCTAGAAATAGCAAGTTAAA |
| P1820 ADH5 KO R | TTGTGTTCTTTCAAGACCATCctcgagGTTGACACTGACGGGATTC |
| P1821 ADH6 KO sgRNA F | GTACCACCACCGCAAAGTAGTTTTAGAGCTAGAAATAGCAAGTTAAA |
| P1822 ADH6 KO R | CTACTTTGCGGTGGTGGTACCctcgagGTTGACACTGACGGGATTC |
| P1823 ADH7 KO sgRNA F | GTATTAGGCCATGAAGGTATTTTTAGAGCTAGAAATAGCAAGTTAAA |
| P1824 ADH7 KO R | ATACCTTCATGGCCTAATACCctcgagGTTGACACTGACGGGATTC |
| P1825 ATF KO sgRNA F | GCTGAAACAGAGTTTCAGCATTTTAGAGCTAGAAATAGCAAGTTAAA |
| P1826 ATF KO R | TGCTGAAACTCTGTTTCAGCCctcgagGTTGACACTGACGGGATTC |
| P1827 KmXDH2 R | CCCAACTTATCTTGAATTGCCctcgagGTTGACACTGACGGGATTC |
| P1833 Adh2 KO Seq F | TTGCCATTGGTCGGTGGTCAC |
| P1834 Adh3 KO Seq F | GAACTTAGCTCAGTCAAGTCCGAA |
| P1839 GlytRNA only F | GCTTTAATTTGCcttCCTAGGgcgGCGGCCGCAATTTCTCTT |
| P1840 GlytRNA only R | cgcCCTAGGaagGCAAATTAAAGCCTTCG |
| P1841 scramble DNA sgRNA F | AGTCCGGTCATTACAACTTATTTTAGAGCTAGAAATAGCAAGTTAAA |
| P1842 tRNA scramble DNA R | TAAGTTGTAATGACCGGACTCctcgagGTTGACACTGACGGGATTC |
| P1850 ATF1 T2 KO sgRNA F | ATATAGTCTTCGGCAACACCTTTTAGAGCTAGAAATAGCAAGTTAAA |
| P1851 ATF1 T2 KO R | GGTGTTGCCGAAGACTATATCctcgagGTTGACACTGACGGGATTC |
| P1857 ADH1 T2 KO sgRNA F | CctcgagGGGCAGCCTGGACAGCGTCAGTTTTAGAGCTAGAAATAGCAAGTTAAA |
| P1858 ADH1 T2 KO R | AAAACTGACGCTGTCCAGGCTGCCCctcgagGTTGACACTGACGGGATTC |
| P1860 ADH2 T2 KO sgRNA F | CctcgagGGTCACCAGCCTTCATTTCAGTTTTAGAGCTAGAAATAGCAAGTTAAA |
| P1861 ADH2 T2 KO R | AAAACTGAAATGAAGGCTGGTGACCctcgagGTTGACACTGACGGGATTC |
| P1868 ADH3 T2 KO sgRNA F | CctcgagGGGACAATTCACAGAATTCACTTTTAGAGCTAGAAATAGCAAGTTAAA |
| P1869 ADH3 T2 KO R | GCTCTAAAAGTGAATTCTGTGAATTGTCCCctcgagGTTGACACTGACGGGATTC |
| P1871 ADH3 T3 KO sgRNA F | CctcgagGGTCACCAGCCTTCAAGCCAGTTTTAGAGCTAGAAATAGCAAGTTAAA |
| P1872 ADH3 T3 KO R | GCTCTAAAACTGGCTTGAAGGCTGGTGACCctcgagGTTGACACTGACGGGATTC |
| P1874 KmADH3 Seq R | AAGGCTTCGGCACCCAATTG |
| P1877 KmAct1Q F | CCCAATGAACCCAAAGAATAACAG |
| P1878 KmAct1Q R | GATAGCATGAGGCAAGGAGAAACC |
| P1879 KmGapdhQ F | GTCCAGAAAGAACATCGAAGTTGTC |
| P1880 KmGapdhQ R | GTAGCTGGGTCTCTTTCTTGGAAG |
| P1932 ADH7 T2 KO sgRNA F | gagGTCTCCTTAGCCATAGCCAAATTTTAGAGCTAGAAATAGCAAGTTAAA |
| P1933 ADH7 T2 KO R | TAAAATTTGGCTATGGCTAAGGAGACctcgagGTTGACACTGACGGGATTC |
| P1937 ADH7 KO Seq R | CCACCTTCCTTGAGAACACG |
| P1938 ADH1 T3 sgRNA F | CctcgagGAGACTTCAAAGCCTTGTACATTTTAGAGCTAGAAATAGCAAGTTAAA |
| P1939 ADH1 T3 R | GCTCTAAAATGTACAAGGCTTTGAAGTCTCctcgagGTTGACACTGACGGGATTC |
| P1941 ADH2 T3 sgRNA F | CctcgagGGGTACCAGCTGGGATGTGAGTTTTAGAGCTAGAAATAGCAAGTTAAA |
| P1942 ADH2 T3 R | GCTCTAAAACTCACATCCCAGCTGGTACCCctcgagGTTGACACTGACGGGATTC |
| P1946 ADH7 T3 sgRNA F | gagGGCTTGAGCTGAGAGATTGATTTTTAGAGCTAGAAATAGCAAGTTAAA |
| P1947 ADH7 T3 R | TAAAAATCAATCTCTCAGCTCAAGCCctcgagGTTGACACTGACGGGATTC |
| P1949 ADH7 KO Seq F | CAACTACATTGAATATAAACATATATATATATCAGC |
| AL30 ADH2 KO Seq R | GACAATGTCCTTAGACTTGGT |
| AL87 KmAdh1 qPCR F | CGTTACTGGCTGGGAAATCG |
| AL88 KmAdh1 qPCR R | GAACCGTCGTGTGTGTAACC |
| AL89 KmAdh2 qPCR F | GTCATCAAGGCTACCAACGG |
| AL90 KmAdh2 qPCR R | CATCGGACTTACACTTGGCG |
| AL91 KmAdh3 qPCR F | GTCCACACGGTGTCATCAAC |
| AL92 KmAdh3 qPCR R | TAACGACGTGGGAGAAGACC |
| AL93 KmAdh4 qPCR F | CAGACCAGCATTACCACCAC |
| AL94 KmAdh4 qPCR R | TCCAACTTACCGCCGTTTTC |
| AL95 KmAdh5 qPCR F | CTTCCACCTCCATTGACTGC |
| AL96 KmAdh5 qPCR R | AACTCAACGCCCTTCAAAGC |
| AL97 KmAdh6 qPCR F | AAGAGATACGGCTGTGGTCC |
| AL98 KmAdh6 qPCR R | ATGGCGTAAACTTCAGCACC |
| AL99 KmAdh7 qPCR F | CGGTGTCCATGGAAAGTCTG |
| AL100 KmAdh7 qPCR R | TGGCAAGCTTTTCGGACTTC |
| AL101 KmAtf1 qPCR F | CTGTCCCCGTTGATGAATCG |
| AL102 KmAtf1 qPCR R | TGGTGTCAATGTGGCCTTAC |
| AL114 FP1 | TATACATGGGATCA TAAATC |
| AL115 RP1 | CTTTGTCTTGTATGATATC |
| AL118 KmAdh1 F | ttttctcttttttacagatcaCCgcGGATGGCTATTCCAGAAACTCAA |
| AL119 KmAdh1 Myc R | AGATAAGTTTTTGTTCacctccgcctagggatccgcctccTTTGGAAGTGTCAACGACAA |
| AL120 KmAdh1 Myc R Extension | ATCTATCGATTTCAATTCAATTCAATACTAGTTTACAGGTCCTCCTCGGAAATCAGCTTTTGTTCacctc |
| AL121 KmAdh2 F | ttttctcttttttacagatcaCCgcGGATGTCTATTCCAACTACTCAAAAGG |
| AL122 KmAdh2 Myc R | GCTTTTGTTCacctccgcctagggatccgcctccTTTGGAAGTGTCAACAACGTAT |
| AL123 KmAdh3 F | ttttctcttttttacagatcaCCgcGGATGCTTAGATTAACTAACGCCAG |
| AL124 KmAdh3 Myc R | GCTTTTGTTCacctccgcctagggatccgcctccTTTTTCAGTGTCGACGACGT |
| AL125 KmAdh4 F | ttttctcttttttacagatcaCCgcGGATGTTCAGACTAGCACGCGC |
| AL126 KmAdh4 Myc R | GCTTTTGTTCacctccgcctagggatccgcctccTTTGGAAGTGTCAACGACGTAT |
| AL127 KmAdh5 F | ttttctcttttttacagatcaCCgcGGATGTTTCATAGAAGAGCATTGAAG |
| AL128 KmAdh5 Myc R | GCTTTTGTTCacctccgcctagggatccgcctccGCATTCATAGGCCTGTCTGA |
| AL129 KmAdh6 F | ttttctcttttttacagatcaCCgcGGATGTCCTACCCAGATAGTTTCC |
| AL130 KmAdh6 Myc R | GCTTTTGTTCacctccgcctagggatccgcctccTTTTTGAGCCTTGAACTCTCC |
| AL131 KmAdh7 F | ttttctcttttttacagatcaCCgcGGATGTTTCGTAAGGTCACATCTG |
| AL132 KmAdh7 Myc R | TTTTGTTCacctccgcctagggatccgcctccAAAGTTAATAATAAGTTTCATAGCCTTT |
| AL133 KmAtf F | ttttctcttttttacagatcaCCgcGGATGATGAAACTACAGAAATTGTCG |
| AL134 KmAtf Myc R | GCTTTTGTTCacctccgcctagggatccgcctccCAATGTAGTCAAGTTGTTTTCAAA |

TCTTTGAAAAGATAATGTATGATTATGCTTTCACTCATATTTATACAGAAACTTGATGTTTTCTTTCGAGTATATACAAGGTGATTACATGTACGTTTGAAGTACAACTCTAGATTTTGTAGTGCCCTCTTGGGCTAGCGGTAAAGGTGCGCATTTTTTCACACCCTACAATGTTCTGTTCAAAAGATTTTGGTCAAACGCTGTAGAAGTGAAAGTTGGTGCGCATGTTTCGGCGTTCGAAACTTCTCCGCAGTGAAAGATAAATGATCAAAGCATTGATCTGCTTTGCTTTTAGAGCTAGAAATAGCAAGTTAAAATAAGGCTAGTCCGTTATCAAGAAAGATAAATGATCCTTGAAAAAGTGGCACCGAGTCGGTGGTGCTTTTTTTGTTTTTTATGTCT

**Figure S7: gblock SNR52-Ade2-sgRNA.** Gblock containing the ScSNR52 promoter (grey shaded), a 20bp Ade2 target sequence (red) and the structural guide RNA for Cas9 recruitment.

CTTGCTCATTAGAAAGAAAGCATAGCAATCTAATCTAAGTTTTCTAGAACTAGTGGATCCCCCGGGaaaaATGGACAAGAAGTACTCTATCGGTTTGGACATCGGTACCAACTCTGTTGGTTGGGCTGTTATCACCGACGAATACAAGGTTCCATCTAAGAAGTTCAAGGTTTTGGGTAACACCGACAGACACTCTATtAAGAAGAAtTTGATCGGTGCTTTGTTGTTCGACTCTGGTGAgACCGCTGAAGCTACCAGATTGAAGAGAACCGCTcGtAGAAGATACACCAGAAGAAAGAACAGAATCTGTTAtTTGCAAGAAATtTTCTCTAACGAgATGGCTAAGGTcGACGAtTCTTTCTTtCACAGATTGGAAGAATCTTTCTTGGTcGAAGAgGAtAAGAAaCACGAAAGACACCCAATCTTCGGTAACATtGTTGACGAAGTTGCTTACCACGAAAAGTACCCAACCATCTACCAtTTGAGAAAaAAGTTGGTTGAtTCcACCGACAAGGCTGACTTGAGATTGATCTACTTGGCcTTGGCcCACATGATCAAGTTCAGAGGTCACTTCTTGATtGAAGGTGACTTGAACCCAGACAAtTCTGACGTTGACAAGTTGTTCATCCAATTGGTTCAAACCTACAACCAATTGTTCGAgGAAAAtCCAATCAACGCTTCcGGTGTcGACGCcAAaGCcATtTTGTCcGCtAGATTaTCTAAaTCTcGtAGATTaGAAAACTTGATtGCcCAATTGCCAGGTGAgAAGAAGAAtGGTTTGTTCGGTAACTTGATCGCcTTGTCcTTGGGTTTaACCCCAAAtTTCAAGTCTAAtTTCGACTTGGCTGAgGAtGCTAAGTTaCAATTGTCcAAGGACACCTACGACGAtGACTTGGAtAACTTaTTGGCTCAAATtGGTGAtCAATACGCcGACTTaTTCTTGGCTGCcAAGAAtTTGTCTGAtGCcATCTTGTTGTCcGAtATCTTGAGAGTTAACACCGAAATCACCAAGGCTCCATTGTCcGCTTCTATGATCAAGAGATACGAtGAACAtCAtCAAGAtTTGACCTTaTTGAAaGCTTTGGTTAGACAACAgTTGCCAGAAAAtTACAAGGAAATtTTCTTtGAtCAATCTAAGAACGGTTAtGCTGGTTACATCGAtGGTGGTGCgTCTCAAGAgGAATTCTACAAGTTCATtAAGCCAATCTTGGAgAAGATGGACGGTACCGAAGAATTGTTGGTTAAGTTGAACAGAGAAGAtTTGTTGAGAAAGCAAAGAACCTTCGACAACGGTTCTATCCCACACCAAATCCACTTGGGTGAATTGCACGCTATCTTGAGAAGACAAGAAGAtTTCTACCCATTCTTGAAGGAtAACAGAGAgAAGATCGAAAAGATtTTGACCTTCAGAATCCCATACTACGTTGGTCCATTGGCTAGAGGTAACTCTAGATTCGCTTGGATGACCAGAAAGTCTGAAGAAACCATCACCCCATGGAACTTCGAAGAAGTcGTTGACAAGGGTGCTTCTGCTCAATCTTTCATCGAAAGAATGACCAACTTCGACAAGAACTTGCCAAACGAAAAGGTTTTGCCAAAGCACTCTTTGTTGTACGAATACTTCACCGTTTACAACGAATTGACCAAGGTTAAGTACGTTACCGAAGGTATGAGAAAGCCAGCTTTCTTGTCTGGTGAACAAAAGAAGGCTATCGTTGACTTGTTGTTCAAGACtAACAGAAAGGTTACCGTcAAGCAgTTGAAGGAAGAtTACTTCAAGAAGATCGAATGTTTCGACTCTGTcGAAATtTCTGGTGTcGAAGACAGATTCAAtGCTTCTTTGGGTACtTACCACGAtTTGTTGAAaATCATtAAGGAtAAGGAtTTCTTaGAtAACGAgGAgAACGAAGAtATtTTGGAgGAtATtGTcTTGACtTTGACtTTGTTtGAgGAtAGAGAAATGATCGAgGAAAGATTGAAGACtTACGCTCAtTTGTTCGAtGAtAAGGTcATGAAGCAATTGAAGAGAAGAAGATACACtGGTTGGGGTAGATTGTCcAGAAAGTTGATCAACGGTATtAGAGAtAAGCAATCcGGTAAGACCATtTTGGAtTTCTTGAAGTCTGACGGTTTCGCTAAtAGAAACTTtATGCAATTGATtCACGACGAtTCTTTGACtTTtAAGGAAGAtATtCAAAAGGCTCAAGTcTCTGGTCAgGGTGAtTCTTTGCACGAACACATCGCTAACTTGGCTGGTTCTCCAGCTATCAAaAAaGGTATtTTGCAAACtGTTAAGGTTGTTGACGAATTGGTTAAGGTTATGGGTAGACACAAGCCAGAgAAtATtGTcATCGAgATGGCTAGAGAAAAtCAAACCACtCAAAAGGGTCAAAAGAACTCcAGAGAgAGAATGAAaAGAATtGAgGAAGGTATCAAGGAgTTGGGTTCcCAAATtTTGAAGGAACACCCAGTcGAgAACACtCAATTGCAAAAtGAAAAGTTGTACTTGTAtTACTTGCAAAACGGTAGAGAtATGTACGTTGACCAAGAATTGGACATtAACAGATTGTCTGACTACGAtGTTGAtCACATCGTTCCACAATCcTTtTTGAAGGAtGACTCTATCGAtAACAAGGTTTTGACtAGATCcGACAAGAAtAGAGGTAAGTCTGAtAACGTTCCATCTGAAGAAGTcGTcAAGAAGATGAAGAACTACTGGAGACAgTTGTTGAAtGCTAAGTTGATtACtCAAAGAAAGTTCGAtAACTTGACtAAGGCTGAgAGAGGTGGTTTGTCTGAgTTGGAtAAGGCcGGTTTCATtAAGAGACAATTGGTcGAAACCAGACAAATCACCAAGCACGTTGCTCAAATCTTGGACTCTAGAATGAACACCAAGTACGACGAAAACGACAAaTTGATtAGAGAgGTTAAGGTcATCACCTTGAAGTCcAAaTTGGTTTCcGACTTtAGAAAGGAtTTCCAATTCTACAAGGTcAGAGAAATcAAtAACTACCACCAtGCTCACGACGCcTACTTGAACGCTGTTGTcGGTACtGCTTTGATcAAGAAGTACCCAAAGTTGGAgTCTGAATTtGTcTACGGTGAtTACAAaGTTTACGACGTTAGAAAGATGATtGCTAAGTCTGAgCAAGAAATCGGTAAGGCTACtGCTAAaTACTTCTTCTACTCcAACATtATGAAtTTCTTCAAGACCGAgATtACCTTGGCcAACGGTGAAATCAGAAAGAGACCATTGATtGAAACtAACGGTGAAACCGGTGAgATtGTcTGGGACAAGGGTAGAGAtTTCGCTACtGTTAGAAAGGTcTTGTCTATGCCACAAGTcAACATtGTcAAGAAGACCGAAGTcCAgACtGGTGGTTTCTCcAAGGAATCTATtTTGCCAAAGAGAAACTCTGAtAAGTTGATtGCcAGAAAGAAaGACTGGGAtCCAAAGAAGTACGGTGGTTTCGACTCTCCAACCGTTGCTTACTCcGTTTTGGTTGTcGCTAAGGTTGAAAAGGGTAAGTCcAAGAAGTTGAAGTCcGTcAAGGAgTTGTTGGGTATtACCATCATGGAAAGATCTTCcTTCGAAAAGAACCCAATtGAtTTCTTGGAgGCTAAGGGTTACAAGGAAGTcAAGAAGGACTTGATCATtAAGTTGCCAAAGTACTCTTTGTTCGAATTGGAgAACGGTAGAAAGAGAATGTTGGCTTCcGCTGGTGAATTGCAAAAGGGTAACGAATTGGCTTTGCCATCTAAGTACGTTAACTTCTTGTACTTGGCTTCcCACTACGAAAAGTTGAAGGGTTCTCCAGAAGAtAACGAACAAAAGCAATTGTTCGTTGAACAACAtAAGCACTACTTGGACGAAATCATCGAACAAATCTCTGAATTCTCTAAGAGAGTTATCTTGGCTGACGCTAACTTGGACAAGGTTTTGTCTGCTTACAACAAGCACAGAGACAAGCCAATCAGAGAgCAAGCTGAAAACATCATCCACTTGTTCACtTTGACCAACTTGGGTGCTCCAGCTGCTTTCAAGTACTTCGACACCACCATCGACAGAAAGAGATACACCTCTACCAAGGAAGTTTTGGACGCTACCTTGATCCACCAATCTATCACCGGTTTGTACGAAACCAGAATCGACTTGTCTCAATTGGGTGGTGACTCTAGAGCTGACCCAAAGAAGAAGAGAAAGGTTTGATCTCTTCTCGAGTCATGTAATTAGTTATGTCACGCTTACATTC

**Figure S8: Codon optimized Cas9 with overlap sequences to plasmid backbone.** The *K. marxianus* codon optimized version of *Sp*Cas9 was manually changed to avoid highly repetitive sequences to allow for production of the gBlocks. The gray sequences represent overlaps to the plasmid backbone for Gibson Assembly cloning.

**
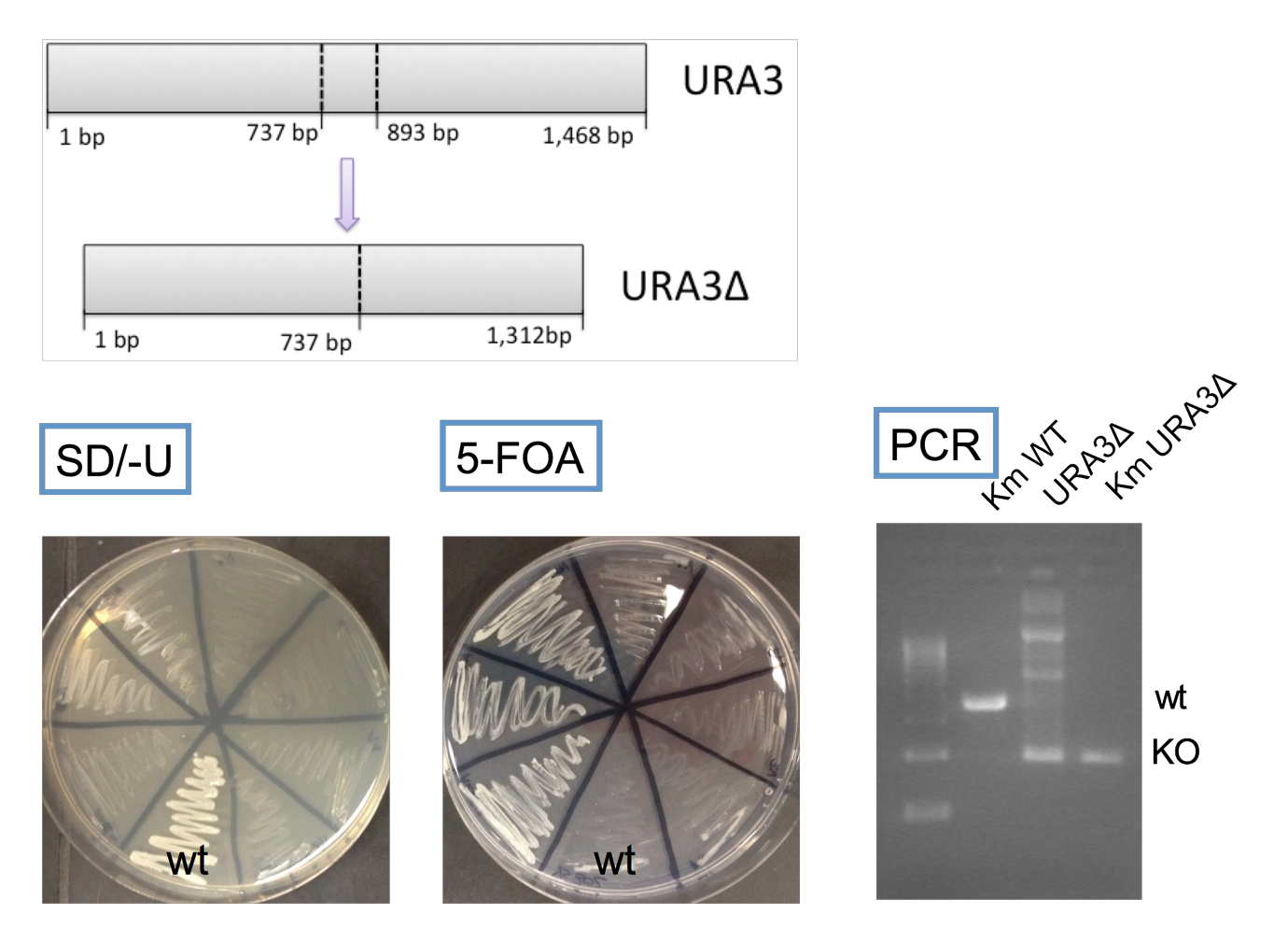
**

**Figure S9: Schematic of URA3 knockout and verification by growth and PCR.** The URA3 knockout was achieved by homologous recombination of a truncated URA3 fragment into the native gene. This leads to a deletion of about 160 bp of the coding region of URA3. Selective pressure for homologous recombination is applied by growing the transformed colonies with the truncated URA3 on agar plates containing 5-fluoroorotic acid (5-FOA). Ability to grow on 5-FOA and inability of growth on SD/-U media indicated URA3 knockout. The knockout was confirmed using primers that bind 100 bp upstream and downstream of the region to be excised so that there is a band of 200 bp for a knockout and 360 bp for the wild type The lanes of the gel show a 100 bp ladder, the wild type URA3 amplicon, as well as the ∆URA3 fragment and the knocked out URA3 gene.

>gi|313024|emb|Z21934.1| K.marxianus URA3 gene

**GAGCATCTTGGTCTTCTGAG**CTCATTATACCTCAATCAAAACTGAAATTAGGTGCCTGTCACGGCTCTTTTTTTACTGTACCTGTGACTTCCTTTCTTATTTCCAAGGATGCTCATCACAATACGCTTCTAGATCTATTATGCATTATAATTAATAGTTGTAGCTACAAAAGGTAAAAGAAAGTCCGGGGCAGGCAACAATAGAAATCGGCAAAAAAAACTACAGAAATACTAAGAGCTTCTTCCCATTCAGTCATCGCATTTCGAAACAAGAGGGGAATGGCTCTGGCTAGGGAACTAACCACCATCGCCTGACTCTATGCACTAACCACGTGACTACATATATGTGATCGTTTTAACATTTTCAAAGGCTGTGTGTCTGGCTGTTTCCATTAATTTTCACTGATTAAGCAGTCATATTGAATCTGAGCTCATCACCAACAAGAAATACTACCGTAAAAGTGTAAAAGTTCGTTTAAATCATTTGTAAACTGGAACAGCAAGAGGAAGTATCATCAGCTAGCCCCATAAACTAATCAAAGGAGGATGTCGACTAAGAGTTACTCGGAAAGAGCAGCTGCTCATAGAAGTCCAGTTGCTGCCAAGCTTTTAAACTTGATGGAAGAGAAGAAGTCAAACTTATGTGCTTCTCTTGATGTTCGTAAAACAGCAGAGTTGTTAAGATTAGTTGAGGTTTTGGGTCCATA**TATCTGTCTATTGAAGACACATGTA**GATATCTTGGAGGATTTCAGCTTTGAGAATACCATTGTGCCGTTGAAGCAATTAGCAGAGAAACACAAGTTTTTGATATTTGAAGACAGGAAGTTTGCCGACATTGGGAACACTGTTAAATTACAATACACGTCTGGTGTATACCGTATCGCCGAATGGTCTGATATC**ACCAATGCACACGGTGTGACTGGTGCGG**GCATTGTTGCTGGTTTGAAGCAAGGTGCCGAGGAAGTTACGAAAGAACCTAGAGGGTTGTTAATGCTTG**CCG**AGTTATCGTCCAAGGGGTCTCTAGCGCACGGTGAATACACTCGTGGGACCGTGGAAATTGCCAAGAGTGATAAGGACTTTGTTATTGGATTTATTGCTCAAAACGATATGGGTGGAAGAGAAGAGGGCTACGATTGGTTGATCATGACGCCAGGTGTTGGTCTTGATGACAAAGGTGATGCTTTGGGACAACAATACAGAACTGTGGATGAAGTTGTTGCCGGTGGATCAGACATCATTATTGTTGGTAGAGGTCTTTTCGCAAAGGGAAGAGATCCTGTAGTGGAAGGTGAGAGATACAGAAAGGCGGGATGGGACGCTTACTTGAAGAGAGTAGGCAGATCCGCTTAAGAGTTCTCCGAGAACATGCAGAGGTTCGAGTGTACTCGGATCAGAAGTTACAAGTTGATCGTTTATATATAAACTATACAGAGATGTTAGAGTGTAATG**GCATTGCGTCACATTGTATAC**

**Figure S10: URA3 knockout fragment** Nucleotide sequence of the *K. marxianus* URA3 gene. The open reading frame of the URA3 gene is shown in blue. The gray shaded area shows the 168bp that are being deleted after overlap PCR. Primers binding sites to create the overlap fragments are shown in bold. Screening primers P1072/1073 are displayed in Table S4

**Table S5: Alcohol dehydrogenases and Alcohol-O-acetyltransferases analyzed for homology to *K. marxianus* proteins**

| **Protein** | **Uniprot Reference** |
| --- | --- |
| *Sc*Adh1 | P00330 |
| *Sc*Adh2 | P00331 |
| *Sc*Adh3 | P07246 |
| *Sc*Adh4 | P10127 |
| *Sc*Adh5 | P38113 |
| *Sc*Adh6 | Q04894 |
| *Sc*Adh7 | P25377 |
| *Cupriavidus necator* adh | P14940 |
| *Snodgrassella alvi* adh | WP_025331133 |
| *Acinetobacter equi* adh | WP_054580671 |
| *Sc*Atf1 | P40353 |
| *Sc*Atf2 | P53296 |
| *Kl*Atf | Q6CJX7 |
| *Kl*Adh1 | P20369 |
| *Kl*Adh2 | P49383 |
| *Kl*Adh3 | P49384 |
| *Kl*Adh4 | P49385 |


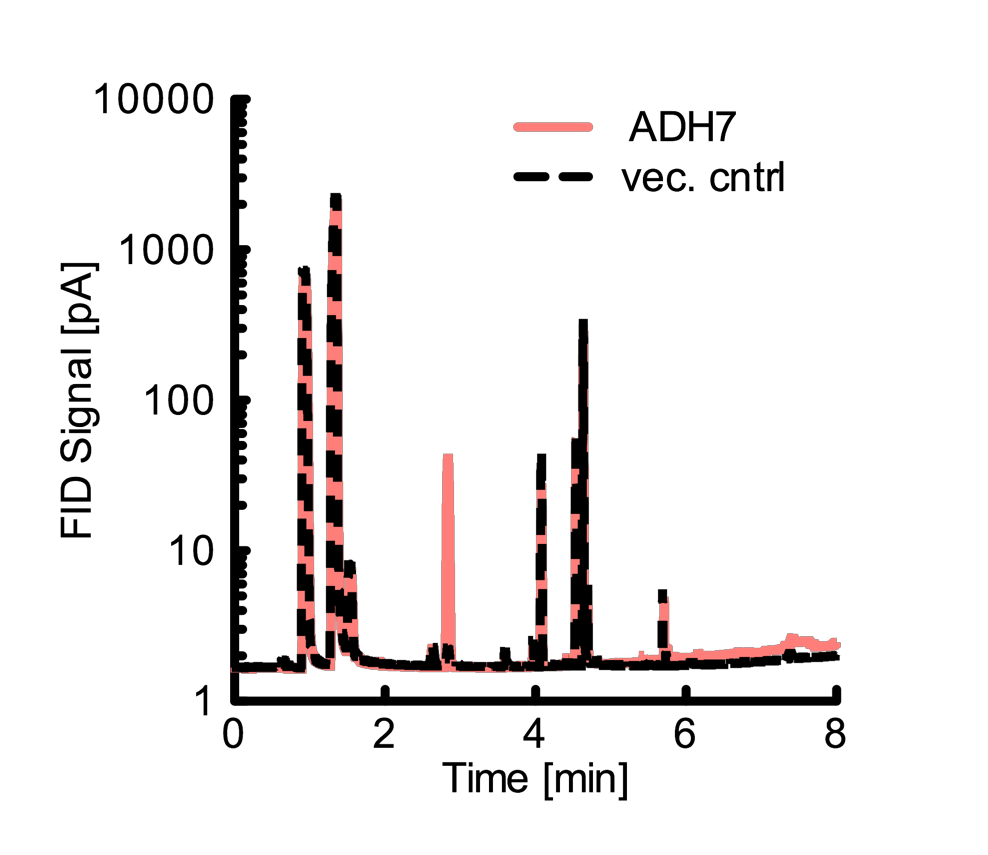


**Figure S11: Comparison of GC chromatograms of the hemiacetal reaction.** The GC samples for *Km*Adh7 overexpression and vector control are shown to confirm the absence of an ethyl acetate peak when the hemiacetal mixture is incubated with protein lacking an alcohol dehydrogenase that is active towards hemiacetal.

SUPPLEMENTAL REFERENCES

1. Lin JL, Wheeldon I: **Dual N- and C-Terminal Helices Are Required for Endoplasmic Reticulum and Lipid Droplet Association of Alcohol Acetyltransferases in Saccharomyces cerevisiae**. *PloS one* 2014, **9**(8).

2. Zhu J, Lin JL, Palomec L, Wheeldon I: **Microbial host selection affects intracellular localization and activity of alcohol-O-acetyltransferase**. *Microbial cell factories* 2015, **14**.

3. DiCarlo JE, Norville JE, Mali P, Rios X, Aach J, Church GM: **Genome engineering in Saccharomyces cerevisiae using CRISPR-Cas systems**. *Nucleic acids research* 2013, **41**(7):4336-4343.

4. Lee KS, Kim JS, Heo P, Yang TJ, Sung YJ, Cheon Y, Koo HM, Yu BJ, Seo JH, Jin YS *et al*: **Characterization of Saccharomyces cerevisiae promoters for heterologous gene expression in Kluyveromyces marxianus**. *Applied microbiology and biotechnology* 2013, **97**(5):2029-2041.
